# Polyunsaturated fatty acids: host-derived antimicrobials against glycolytic *Staphylococcus aureus*

**DOI:** 10.1128/jb.00249-26

**Published:** 2026-09-17

**Authors:** Alex R. Stackhouse, Asif Iqbal, D. Grace Ross, Wyatt W. Wittliff, Kirsten E. Rico, Bennett J. Blank, Brooke M. LeBreton, Sara P. Bucher, Michelle Y. MacIntyre, William N. Beavers

**Affiliations:** 1 Department of Pathobiological Sciences, Louisiana State University and Agricultural and Mechanical College, School of Veterinary Medicine, Baton Rouge, Louisiana, USA; 2 Louisiana State University School of Veterinary Medicine Mass Spectrometry Resource Center, Louisiana State University and Agricultural and Mechanical College, School of Veterinary Medicine, Baton Rouge, Louisiana, USA; Southern University of Science and Technology, Shenzhen, Guangdong, China

**Keywords:** *Staphylococcus aureus*, polyunsaturated fatty acids, arachidonic acid, small colony variants, glycolysis, persisters, lipid peroxidation, lipid electrophiles

## Abstract

Metabolic flexibility allows *Staphylococcus aureus* to survive killing by antibiotics and antimicrobial immune molecules while infecting every niche of the mammalian host. Whether *S. aureus* is using glycolysis, aerobic respiration, or anaerobic respiration to generate energy, these cellular processes must be accurately attuned to the infected host tissue. Shifting from respiration to glycolysis lowers metabolic throughput, allowing the transition to a persistent *S. aureus* infection and avoiding killing by most antimicrobial molecules, which typically target rapidly dividing cells. Previously, we reported that arachidonic acid (AA), an abundant host polyunsaturated fatty acid (PUFA), kills *S. aureus* through a lipid peroxidation mechanism. Here, we report that the extent of PUFA killing of *S. aureus* can be predicted by the autoxidation rate constant, extending our previous AA findings to include all PUFAs. Contrasting many other antimicrobial molecules, AA kills glycolytic *S. aureus* more effectively than respiring *S. aureus*. Elevating cellular ATP levels protects *S. aureus* from PUFA killing, indicating that glycolytic *S. aureus* strains cannot sufficiently energize the ATP-dependent processes that allow *S. aureus* to survive PUFA toxicity. This report defines cellular metabolism as a key determinant in *S. aureus* PUFA resistance and demonstrates that PUFAs are effective antimicrobials against glycolytic *S. aureus*, including persister cells and small colony variants. Furthermore, identifying and validating the ATP-dependent processes that protect *S. aureus* from PUFA killing will define pathways that can be targeted to accentuate killing by the host immune system, leading to the clearance of persistent *S. aureus* infections.

**IMPORTANCE:**

*Staphylococcus aureus* causes significant human morbidity and mortality. The metabolic diversity of this pathogen makes eradication challenging, leading to persistent infections in many patient populations. Polyunsaturated fatty acids (PUFAs) are host-derived antimicrobial molecules that are abundant at the host-pathogen interface. Here, we define the mechanism of killing by PUFAs in metabolically limited *S. aureus* strains. These strains are difficult to eradicate because most therapies target rapidly dividing cells. We demonstrate that PUFAs kill slow-growing *S. aureus* through a lipid peroxidation mechanism better than their normal growth counterparts. Restoring ATP levels in these strains protects against PUFA killing, demonstrating that the PUFA survival response is not fully energized in the low metabolic state.

## INTRODUCTION

*Staphylococcus aureus* can colonize the skin of nearly two in three people (1). While *S. aureus* can live on the skin of humans for decades without causing infection, its transition from commensal to pathogen has dire consequences for human morbidity and mortality. In 2019, *S. aureus* was associated with an estimated 1.1 million deaths worldwide (2). The high impact of *S. aureus* on human health is not simply due to its prevalence, but also to its ability to infect every niche of the human host regardless of nutrient availability or oxygen concentration (3). Broad infectivity is enabled in *S. aureus* due to diverse and tunable metabolic programs that can generate energy under a panoply of host conditions. *S. aureus* can consume various carbon sources, including multiple sugars (4), amino acids (5), and even pyruvate directly (6), which can be converted into energy through glycolysis, aerobic respiration, and anaerobic respiration (7).

Persister cells are metabolically dormant *S. aureus* (8), characterized by tolerance toward antibiotics (9, 10), oxidative stressors (11, 12), and the host immune system (12, 13). Persister cells can revert to standard growth phenotypes, so small colony variants (SCVs) can be used as models to study some persister cell phenotypes, such as antibiotic tolerance (14). SCVs have decreased cellular metabolism and growth due to stable genetic mutations, causing a smaller colony size and serving as the origin for their descriptive name (14). Both persister cells and SCVs are important during *S. aureus* infection as they can be a reservoir for refractory and persistent infections (15–17). As such, much work has been done by us and others to better understand how metabolism contributes to persistence and to discover mechanisms that restore sensitivity to antimicrobial therapies (9, 10, 15).

Polyunsaturated fatty acids (PUFA) are abundant at the host-pathogen interface (18). They are not present in *S. aureus* basally (19) but are released from the lipid bilayer of host immune cells in response to cellular damage and pathogen infection as the precursors to lipid inflammatory mediators (18, 20). PUFAs not only kill *S. aureus* (19), but also other bacterial pathogens as well (21–24). Unsaturated fatty acids disrupt membrane integrity of *S. aureus,* allowing equilibration of molecules across the membrane (9, 19, 25–27). We exploited this phenomenon to resensitize antibiotic-tolerant *S. aureus* to aminoglycoside antibiotics when co-treated with PUFAs (9). Unlike monounsaturated fatty acids, PUFAs contain multiple *cis* double bonds separated by a bis-allylic carbon that makes them susceptible to oxidation (28). We were the first to report that the mechanism of killing by arachidonic acid (AA) is through a lipid peroxidation mechanism where AA is oxidized to lipid electrophiles that form protein post-translational modifications (PTMs). When these PTMs accumulate, the *S. aureus* cell dies (19). While it was previously known that lipid peroxidation of PUFAs can kill mammalian cells, this was the first report, to our knowledge, that lipid peroxidation can be exploited as an antimicrobial mechanism.

Since SCVs can tolerate higher levels of most antimicrobial compounds, compared to wild-type *S. aureus*, we tested the central hypothesis that the oxidants generated during respiration drive antimicrobial lipid peroxidation and that inactivating the electron transport chain (ETC) will result in SCVs with increased resistance to PUFA killing. Here, we report that the previously observed PUFA killing phenotypes are mediated exclusively through autoxidation or non-enzymatic lipid peroxidation. Contrary to our initial hypothesis, SCVs and persister cells are more sensitive to PUFA killing than *S. aureus* strains that generate energy through oxidative phosphorylation. This increased killing is also observed under anaerobic conditions where the ETC is inactivated by the depletion of the canonical terminal electron acceptor, molecular oxygen. The killing phenotypes inversely correlate with ATP levels and can be reversed by increasing cellular ATP, implying that one or more ATP-dependent processes are responsible for the observed protection. We report that lipid peroxidation and the resulting lipid electrophile PTMs are a potential antimicrobial mechanism to eliminate persistent and refractory *S. aureus* infections that have long been an elusive target in *S. aureus* antimicrobial resensitization.

## RESULTS

### Polyunsaturated fatty acids kill *S. aureus* through an autoxidation mechanism

**Fig 1:**
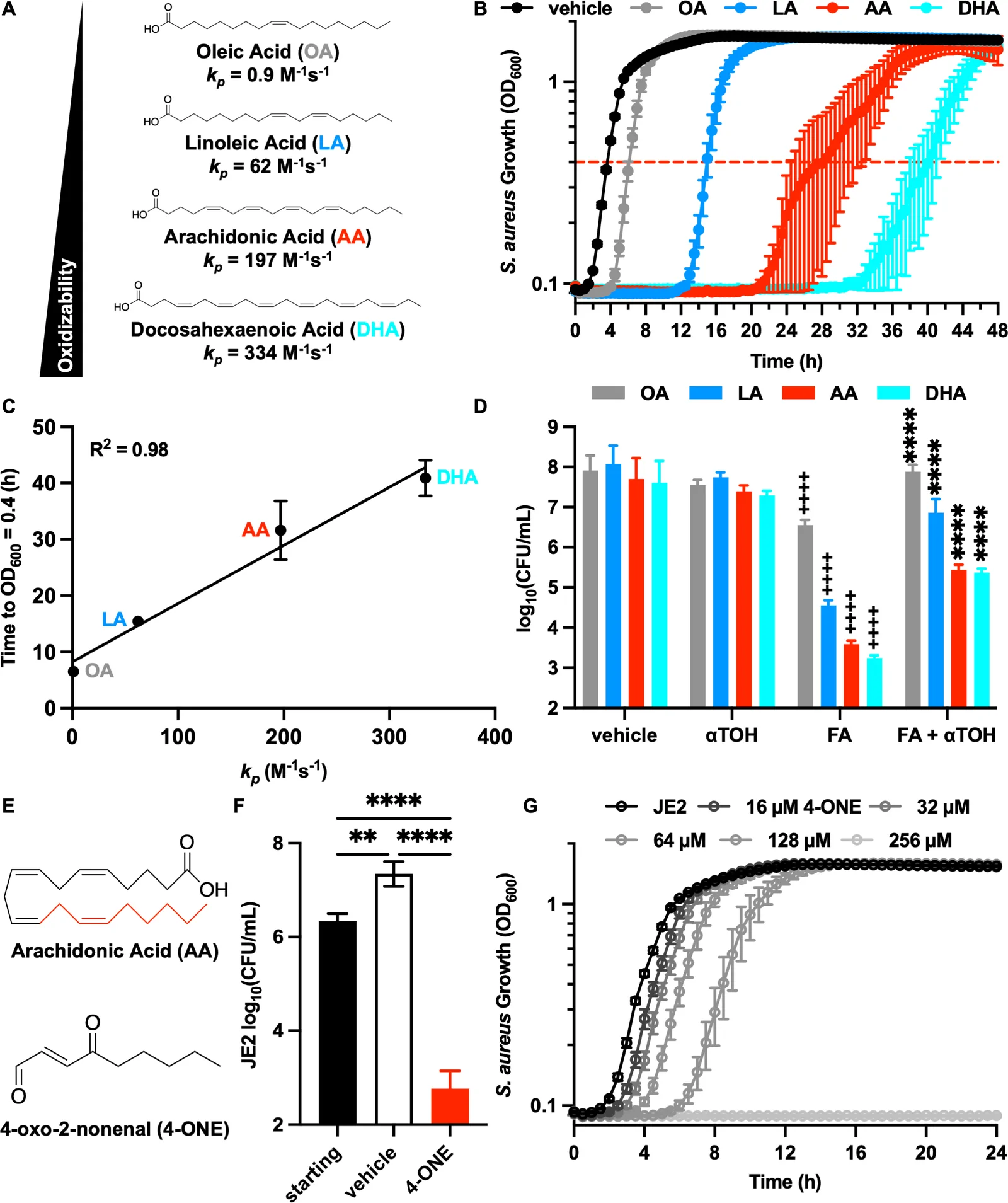
PUFA killing of *S. aureus* is mediated through autoxidation. (**A**) Structures and *k*_*p*_ values for OA, LA, AA, and DHA. (**B**) JE2 overnights were diluted and treated with or without 100 μM OA, LA, AA, or DHA. Growth was monitored by optical density at 600 nm for 48 h. Data are mean ± S.E.M. for measurements acquired for six biological replicates per condition. (**C**) The time for each curve in Panel** B** to reach OD_600_ = 0.4 (dashed red line) compared to the *k*_*p*_ for each fatty acid was plotted. Simple linear regression calculated the *R*^2^ value to determine the correlation between fatty acid-induced growth delays and the *k*_*p*_ for each fatty acid. Data are mean ± S.D. for six biological replicates per condition. (**D**) JE2 was treated with or without OA, LA, AA, or DHA and incubated shaking at 37°C for 4 h. CFUs were enumerated by dilution plating onto solid medium. Data are mean ± S.D. acquired in biological triplicate. Statistical significance was determined by two-way ANOVA where “+” delineates comparisons between FA-treated JE2 and untreated JE2 and “*” delineates comparisons between FA and αTOH-treated JE2 and FA-treated JE2. ++++ and **** = *P* < 0.0001. (**E**) The structures of AA and an AA-derived lipid electrophile, 4-oxo-2-nonenal (4-ONE). The red portion of AA can be converted into 4-ONE during lipid peroxidation. (**F**) JE2 overnights were diluted and treated with 256 μM 4-ONE for 3 h. Killing was assessed by dilution plating onto solid medium following the incubation. Data are mean ± S.D. for data acquired in biological quadruplicate. Statistical significance was determined by one-way ANOVA, where ** = *P* < 0.01 and **** = *P* < 0.0001. (**G**) JE2 overnights were diluted and treated with a range of 4-ONE concentrations. Growth was monitored by optical density at 600 nm for 24 h. Data are mean ± S.E.M. for measurements acquired in biological triplicate.

The structure of unsaturated fatty acids, specifically PUFAs, makes them susceptible to oxidation in the cellular milieu. Oxidation proceeds through abstraction of a hydrogen atom from the bis-allylic carbon of PUFAs (28). Figure 1A shows the structures of abundant mammalian fatty acids, including monounsaturated oleic acid (OA) and PUFAs linoleic acid (LA), arachidonic acid (AA), and docosahexaenoic acid (DHA), and their respective rate constants for the lipid peroxidation autoxidation propagation reaction (*k*_*p*_) (29). The ability of each of these fatty acids to inhibit *S. aureus* USA300 JE2 (JE2) viability was assessed by kinetic growth. Each fatty acid induces a delay in growth proportional to the number of double bonds it contains (Fig. 1B). The *k*_*p*_ is a measure of how susceptible a fatty acid is to autoxidation (Fig. S1) (29), and plotting the *k*_*p*_ for each fatty acid against the growth delay caused by that fatty acid (Fig. 1B) demonstrates a linear correlation between these two values (Fig. 1C). This linear correlation confirms our previous observation that PUFAs kill *S. aureus* through a lipid peroxidation mechanism (19) and identifies autoxidation or non-enzymatic oxidation of PUFAs as the driver of the killing phenotype. If enzymatic oxidation were involved in this process, one or multiple of the PUFAs tested would display enzymatic substrate preference. Figure 1B identifies a growth delay but does not determine if growth is arrested or if the cells are being killed by PUFA treatment. Dilution plating for CFU enumeration (Fig. 1D) demonstrates that PUFAs kill *S. aureus* proportional to their unsaturation and that α-tocopherol (αTOH), an antioxidant that halts lipid peroxidation (Fig. S1), protects *S. aureus* from killing by all fatty acids tested. In total, these data confirm our previously reported killing phenotypes for AA (19) and demonstrate applicability across all PUFAs that autoxidation kinetics predict bactericidal activity against *S. aureus*.

### Exogenous lipid electrophiles dose-dependently inhibit *S. aureus* growth

PUFA killing of *S. aureus* requires cellular uptake, oxidation through lipid peroxidation to lipid electrophiles, and then toxic PTM formation by the lipid electrophiles (Fig. 1) (19). The lipid electrophiles generated within the cell are termed endogenous lipid electrophiles (30). Due to the oxidizing nature of the host-pathogen interface, *S. aureus* will likely experience high levels of exogenous lipid electrophiles as well, with one example being 4-oxo-2-nonenal (4-ONE) (31). Figure 1E shows the structure of AA and the structure of 4-ONE with the portion of AA that is converted into 4-ONE during lipid peroxidation annotated in red. We tested the ability of 4-ONE to kill *S. aureus* (Fig. 1F) and observed that exogenous lipid electrophiles are dose-dependently toxic against *S. aureus* as well (Fig. 1G).

### PUFAs induce metabolic alterations in *S. aureus* through membrane disruption

**Fig 2:**
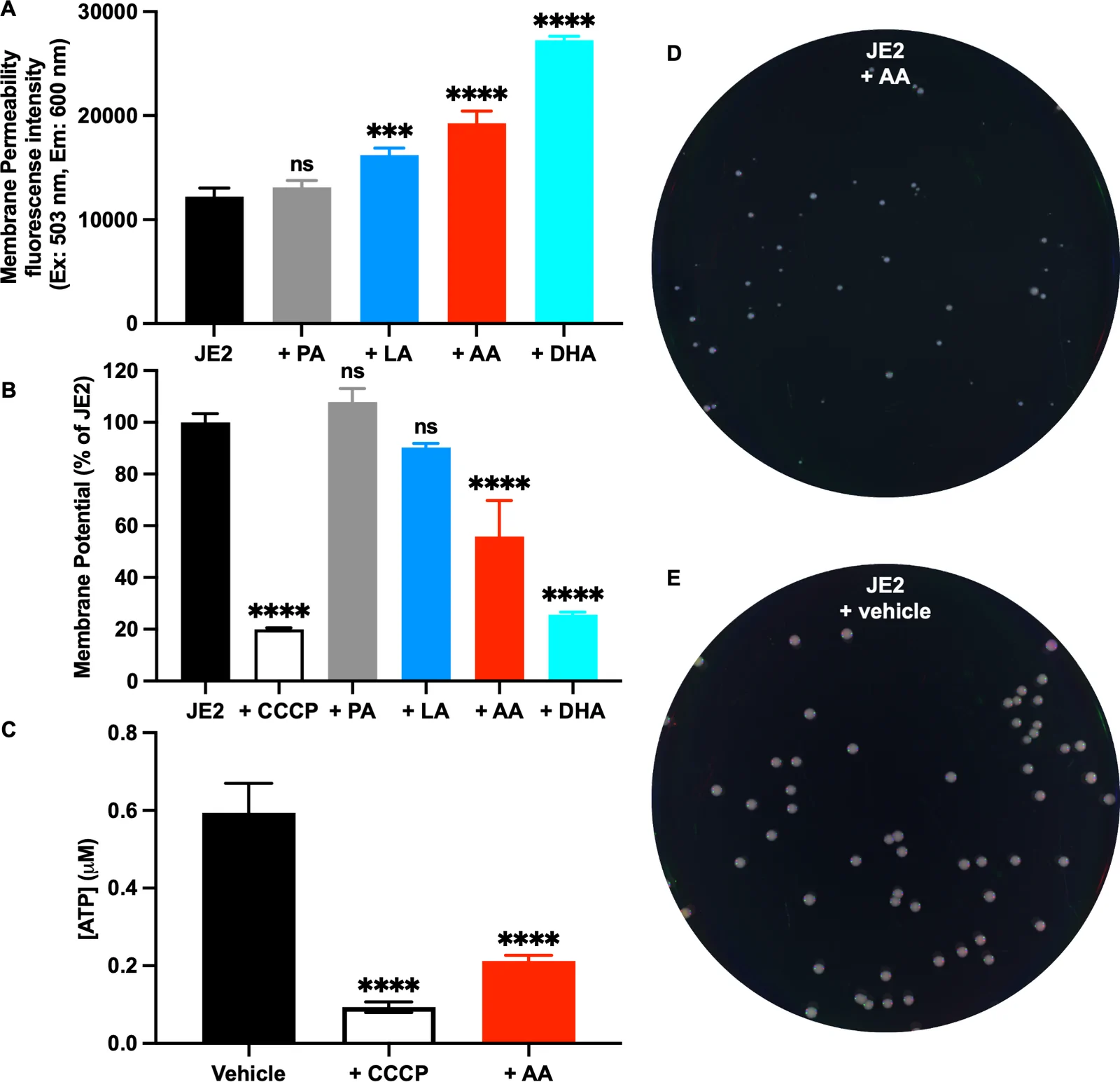
PUFAs disrupt the *S. aureus* membrane, altering cellular metabolism. (**A**) Membrane permeability was assessed by combining JE2 with or without PA, LA, AA, or DHA and ethidium bromide. Fluorescence measurements (ex: 503 nm, em: 600 nm) monitored the ability of ethidium bromide to enter the cell. Data are mean ± S.D. for data acquired in biological triplicate. Statistical significance was determined by one-way ANOVA, where ns = *P* > 0.05, *** = *P* < 0.001, and **** = *P* < 0.0001. (**B**) Membrane potential was assessed in JE2 treated with or without CCCP, PA, LA, AA, or DHA. Fluorescence measurements (red, ex: 488 nm, em: 595 nm; green, ex: 488 nm, em: 530 nm) monitored the ratio of the dye inside and outside of the cell. Data are mean ± S.D. acquired in biological triplicate. Statistical significance was determined by one-way ANOVA, where ns = *P* > 0.05 and **** = *P* < 0.0001. (**C**) Cellular ATP levels were measured in JE2 treated with or without CCCP or AA. Data are mean ± S.D. acquired in biological triplicate. Statistical significance was determined by one-way ANOVA, where **** = *P* < 0.0001. JE2 overnights were diluted and plated onto tryptic soy agar (TSA) with (**D**) and without (**E**) AA and grown overnight at 37°C to compare colony size following AA treatment.

We previously demonstrated that AA disrupts the *S. aureus* membrane due to the *cis* configuration of the double bonds, leading to increased membrane permeability (19). We tested the hypothesis that membrane permeability will be altered by the number of double bonds each PUFA contains. Figure 2A demonstrates that palmitic acid (PA), a saturated fatty acid, does not alter membrane permeability. In contrast, the PUFAs LA, AA, and DHA increase membrane permeability in correlation with the number of *cis* double bonds contained within each PUFA. The observed increase in membrane permeability from unsaturation results in a concomitant decrease in membrane potential (Fig. 2B) and intracellular ATP concentrations (Fig. 2C). The membrane depolarizing compound carbonyl cyanide *m*-chlorophenylhydrazone (CCCP) was used as a control for complete membrane depolarization (32). These data are consistent with previous work showing that unsaturated fatty acids cause the leakage of intracellular contents from *S. aureus* (25–27). Also, consistent with the loss of membrane potential and the decrease in intracellular ATP, *S. aureus* grown on solid medium containing AA has a smaller colony size (Fig. 2D) compared to the same strain grown on solid medium alone (Fig. 2E). These data demonstrate that PUFAs disrupt the *S. aureus* membrane, altering cellular ATP production.

### Bicarbonate repolarizes the *S. aureus* membrane, protecting the pathogen from AA killing

We and others described that bicarbonate, one of the most abundant molecules in mammalian blood and body fluids, hyperpolarizes the *S. aureus* membrane (33, 34). Consistent with previous findings, bicarbonate increases the membrane potential of JE2, JE2 treated with CCCP, and JE2 treated with AA (Fig. S2A). Membrane hyperpolarization results in an increase in intracellular ATP in JE2 treated with bicarbonate and AA compared to AA treatment alone (Fig. S2B). The restoration of membrane potential and intracellular ATP levels protects JE2 from AA killing (Fig. S2C), correlating ATP levels and the metabolic state of the cell with AA susceptibility.

### Menaquinone biosynthesis provides resistance to AA killing

**Fig 3:**
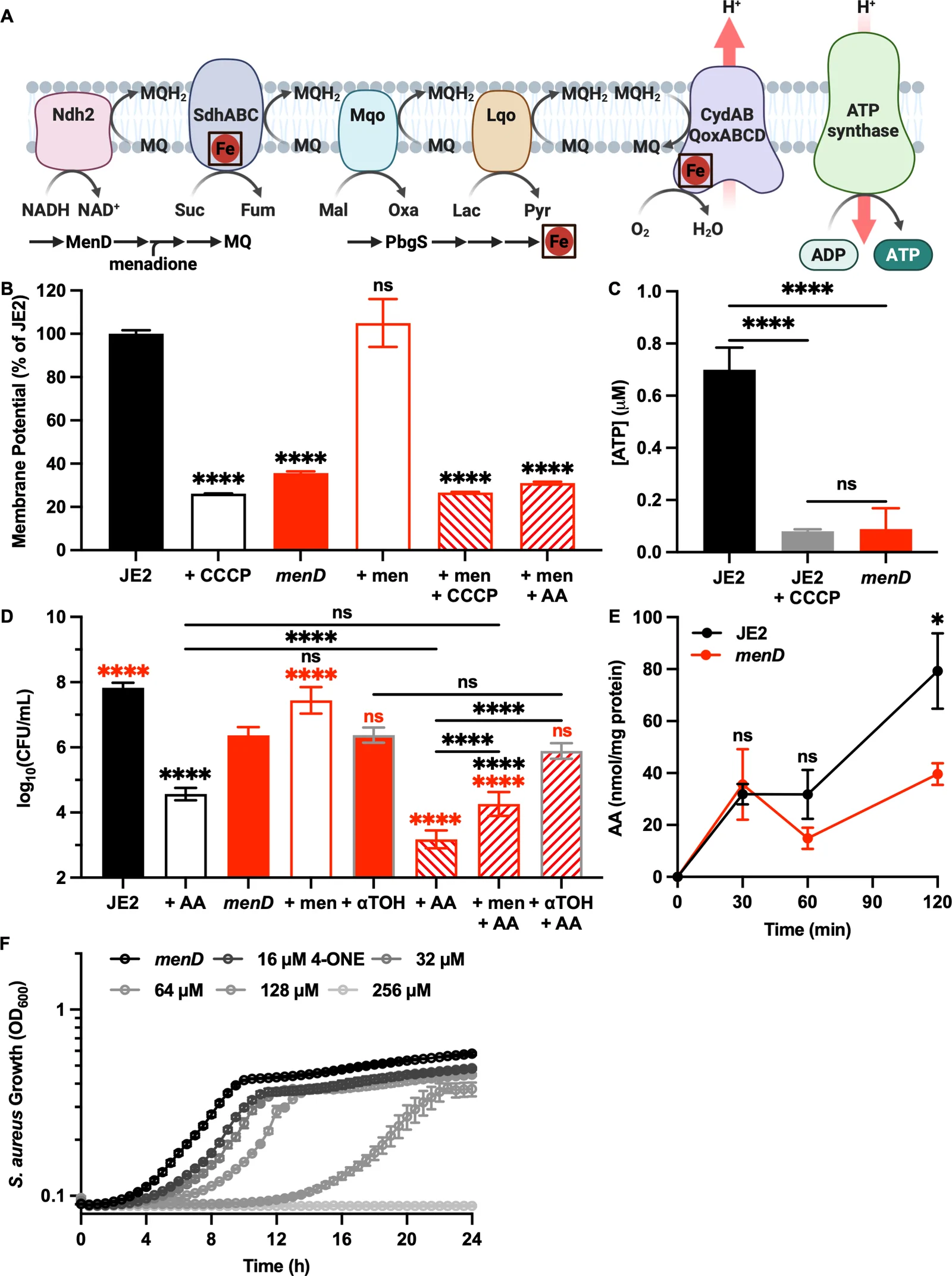
The *S. aureus* SCV, *menD*, is highly susceptible to AA killing. (**A**) The *S. aureus* aerobic ETC with all mutated pathways studied in this manuscript annotated. (**B**) Membrane potential was assessed in JE2 treated with or without CCCP or in *menD* treated with and without CCCP, menadione (men), and AA. Fluorescence measurements (red, ex: 488 nm, em: 595 nm; green, ex: 488 nm, em: 530 nm) monitored the ratio of the dye inside and outside of the cell. Data are mean ± S.D. acquired in biological triplicate. Statistical significance was determined by one-way ANOVA, where ns = *P* > 0.05 and **** = *P* < 0.0001. (**C**) Cellular ATP levels were measured in JE2 or *menD* treated with or without CCCP. Data are mean ± S.D. acquired in biological triplicate. Statistical significance was determined by one-way ANOVA, where ns = *P* > 0.05 and **** = *P* < 0.0001. (**D**) JE2 or *menD* was combined with or without AA, menadione (men), or αTOH and incubated for 4 h. Killing was assessed by dilution plating onto solid medium following the incubation. Data are mean ± S.D. for six biological replicates per condition. Statistical significance was determined by one-way ANOVA, where ns = *P* > 0.05 and **** = *P* < 0.0001. Black stars indicate the comparison to JE2 (solid black bar), while red stars indicate the comparison to *menD* (solid red bar). All other comparisons are denoted by lines on the graph. (**E**) JE2 and *menD* were treated with AA and incubated for various time points. Lipids were extracted and base hydrolyzed to free fatty acids before being quantified by LC-MS/MS. Data are mean ± S.D. acquired in biological quadruplicate, normalized to total protein. Statistical significance was determined by *t*-test, where ns = *P* > 0.05 and * = *P* < 0.0001. (**F**) *menD* overnights were diluted and treated with a range of 4-ONE concentrations. Growth was monitored by optical density at 600 nm for 24 h. Data are mean ± S.E.M. for measurements acquired in biological triplicate.

*S. aureus* will form persister cells in response to many cellular stressors, including antibiotics (9, 10), oxidative stress (11, 12), and serum (13). Persister cells enter a low metabolic state, similar to what we observed in Fig. 2, allowing them to survive antimicrobial stressors and restore virulence when the stress is resolved (8). Persister cells are difficult to study due to their ability to shift metabolism, so we use SCVs as a metabolically stable model system (35–37). SCVs have genetic mutations that prevent the cell from running oxidative phosphorylation, allowing us to model persister cell metabolism (35, 37, 38). The first SCV strain we tested was *S. aureus* USA300 JE2 Δ*menD* (*menD*) that has *menD* deleted, which encodes an essential menaquinone biosynthesis enzyme (35). This mutation is clinically relevant as strains containing *menD* inactivating mutations have been isolated from patient populations (35, 36, 39). Menaquinone is the electron carrier of the *S. aureus* ETC, where menaquinone is reduced to menaquinol by the NADH:menaquinone oxidoreductase, succinate dehydrogenase, malate-quinone oxidoreductase, and lactate-quinone oxidoreductase complexes (Fig. 3A) (35). The terminal oxidases of *S. aureus* oxidize menaquinol to menaquinone, transferring those electrons to molecular oxygen, the preferred aerobic terminal electron acceptor (Fig. 3A) (38). Deleting *menD* results in a non-functional ETC and thus a decrease in membrane potential (Fig. 3B) and intracellular ATP (Fig. 3C). Membrane potential is rescued by the addition of exogenous menadione, which is used in menaquinone biosynthesis upstream of MenD (40), restoring ETC function (Fig. 3B). Dilution plating determined that *menD* is killed 10-fold less by AA when it is chemically complemented with menadione, comparable to JE2 (Fig. 3D), indicating that *menD* is more susceptible to AA killing than JE2. The antioxidant, α-tocopherol (41), completely protects *menD* from AA killing, demonstrating that AA kills SCVs through a lipid peroxidation mechanism as well (Fig. 3D). The *menD* strain has less intracellular AA compared to JE2, as determined by LC-MS/MS, demonstrating that the increased AA susceptibility in *menD* is not due to increased AA uptake (Fig. 3E and Table S1). The deletion of *menD* also sensitizes *S. aureus* to exogenous lipid electrophiles (Fig. 3F).

To further test our previous findings in *menD* that PUFAs kill *S. aureus* through an autoxidation lipid peroxidation mechanism (Fig. 3D), we measured the ability of LA, AA, and DHA to inhibit *menD* growth. Due to SCV susceptibility, the concentration of each PUFA had to be decreased to avoid complete killing. Kinetic growth measurements confirm that *menD* is more susceptible to PUFAs than JE2 (Fig. S3A). Additionally, we plotted the time for *menD* to reach OD_600_ = 0.13 for each PUFA against the *k*_*p*_ for each PUFA. Figure S3B demonstrates a linear relationship between the growth delay and the autoxidation propagation rate constant, confirming that PUFAs undergo autoxidation in SCVs as well. In total, these data demonstrate that *menD* is more susceptible to PUFA killing compared to the parental JE2 strain and that the mechanisms of PUFA oxidation are the same in SCVs and JE2.

### A functional ETC protects *S. aureus* from PUFA killing

**Fig 4:**
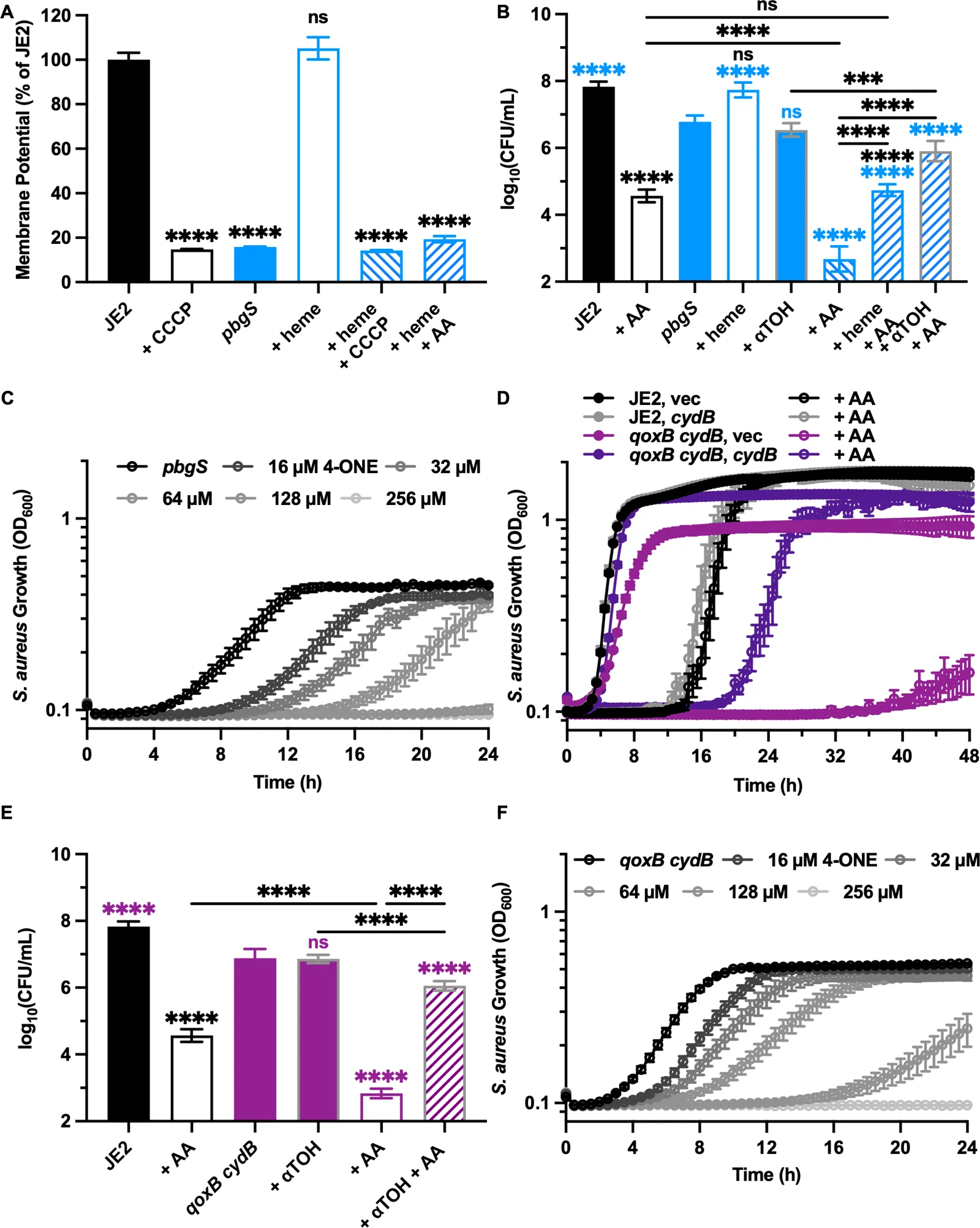
ETC alterations sensitize *S. aureus* SCVs to AA killing. (**A**) Membrane potential was assessed in JE2 treated with or without CCCP or in *pbgS* treated with and without CCCP, heme, and AA. Fluorescence measurements (red, ex: 488 nm, em: 595 nm; green, ex: 488 nm, em: 530 nm) monitored the ratio of the dye inside and outside of the cell. Data are mean ± S.D. acquired in biological triplicate. Statistical significance was determined by one-way ANOVA, where ns = *P* > 0.05 and **** = *P* < 0.0001. (**B**) JE2 or *pbgS* was combined with or without AA, heme, or αTOH and incubated for 4 h. Killing was assessed by dilution plating onto solid medium following the incubation. Data are mean ± S.D. for six biological replicates per sample. Statistical significance was determined by one-way ANOVA, where ns = *P* > 0.05, *** = *P* < 0.001, and **** = *P* < 0.0001. Black stars indicate the comparison to JE2 (solid black bar), while blue stars indicate the comparison to *pbgS* (solid blue bar). All other comparisons are denoted by lines on the graph. (**C**) *pbgS* overnights were diluted and treated with a range of 4-ONE concentrations. Growth was monitored by optical density at 600 nm for 24 h. Data are mean ± S.E.M. for measurements acquired in biological triplicate. (**D**) JE2 or *qoxB cydB* harboring the empty vector plasmid (vec) or constitutively expressing *cydB* in *trans* were treated with or without AA. Growth was monitored by optical density at 600 nm for 48 h. Data are mean ± S.E.M. for measurements acquired in biological quadruplicate. (**E**) JE2 or *qoxB cydB* was combined with or without AA or αTOH and incubated for 4 h. Killing was assessed by dilution plating onto solid medium following the incubation. Data are mean ± S.D. for six biological replicates per condition. Statistical significance was determined by one-way ANOVA, where ns = *P* > 0.05 and **** = *P* < 0.0001. Black stars indicate the comparison to JE2 (solid black bar), while purple stars indicate the comparison to *qoxB cydB* (solid purple bar). All other comparisons are denoted by lines on the graph. (**F**) *qoxB cydB* overnights were diluted and treated with a range of 4-ONE concentrations. Growth was monitored by optical density at 600 nm for 24 h. Data are mean ± S.E.M. for measurements acquired in biological triplicate.

We tested if the PUFA susceptibility phenotype observed for *menD* is specific to the loss of menaquinone biosynthesis or if that discovery can be applied broadly to SCV strains. The next SCV tested is *S. aureus* USA300 JE2 *pbgS::Tn* (*pbgS*), a strain with *pbgS*, a necessary enzyme in heme biosynthesis, inactivated (37). Heme is a critical enzymatic cofactor for many cellular processes, including the function of the ETC enzyme complexes succinate dehydrogenase and the terminal oxidases (Fig. 3A) (38). This mutation is clinically relevant as strains containing *pbgS* inactivating mutations have been isolated from patient populations (16). Inactivation of *pbgS* ablates membrane potential, confirming that the ETC is not functional (Fig. 4A). Membrane potential is not further decreased with CCCP or AA, but is rescued with the addition of exogenous heme, demonstrating that the phenotype is specific to the inactivation of *pbgS* (Fig. 4A). Inactivating *pbgS* is more bactericidal than inactivating *menD*, with *pbgS* being over 300-fold more susceptible to AA than JE2 or the chemically complemented *pbgS* strain (Fig. 4B). The antioxidant, α-tocopherol, protects *pbgS* from AA killing, confirming that the mechanism of killing is through lipid peroxidation in this SCV as well. The *pbgS* strain is also dose-dependently susceptible to the exogenous lipid electrophile, 4-ONE (Fig. 4C).

Both menaquinone and heme are essential cofactors for ETC function but also have other roles in the *S. aureus* cell. *S. aureus* has redundant terminal oxidase complexes (QoxABCD and CydAB) (38). Therefore, we inactivated a gene encoding an essential enzyme for each complex to derive the ETC-specific knockout strain, *S. aureus* USA300 JE2 *qoxB::Tn* Δ*cydB* (*qoxB cydB*). A kinetic growth curve shows that *qoxB cydB* has a growth defect compared to JE2 that can be complemented in *trans* through the constitutive expression of *cydB* (Fig. 4D). The *qoxB cydB* strain is more susceptible to AA toxicity than either JE2 or the *cydB* complemented strain, demonstrating that specific inactivation of the ETC sensitizes *S. aureus* to AA killing (Fig. 4D). Dilution plating quantifies that *qoxB cydB* is killed more than JE2 by AA and is protected from AA killing by α-tocopherol, confirming that the observed killing is lipid peroxidation mediated (Fig. 4E). Finally, *qoxB cydB* is dose-dependently susceptible to exogenous lipid electrophiles (Fig. 4F). In total, these data identify inactivation of the ETC and the resulting cellular metabolic changes as determinants in *S. aureus* sensitization to PUFA killing.

### The redox cycling herbicide paraquat induces persister-like phenotype in *S. aureus* and sensitizes *S. aureus* to PUFA killing

**Fig 5:**
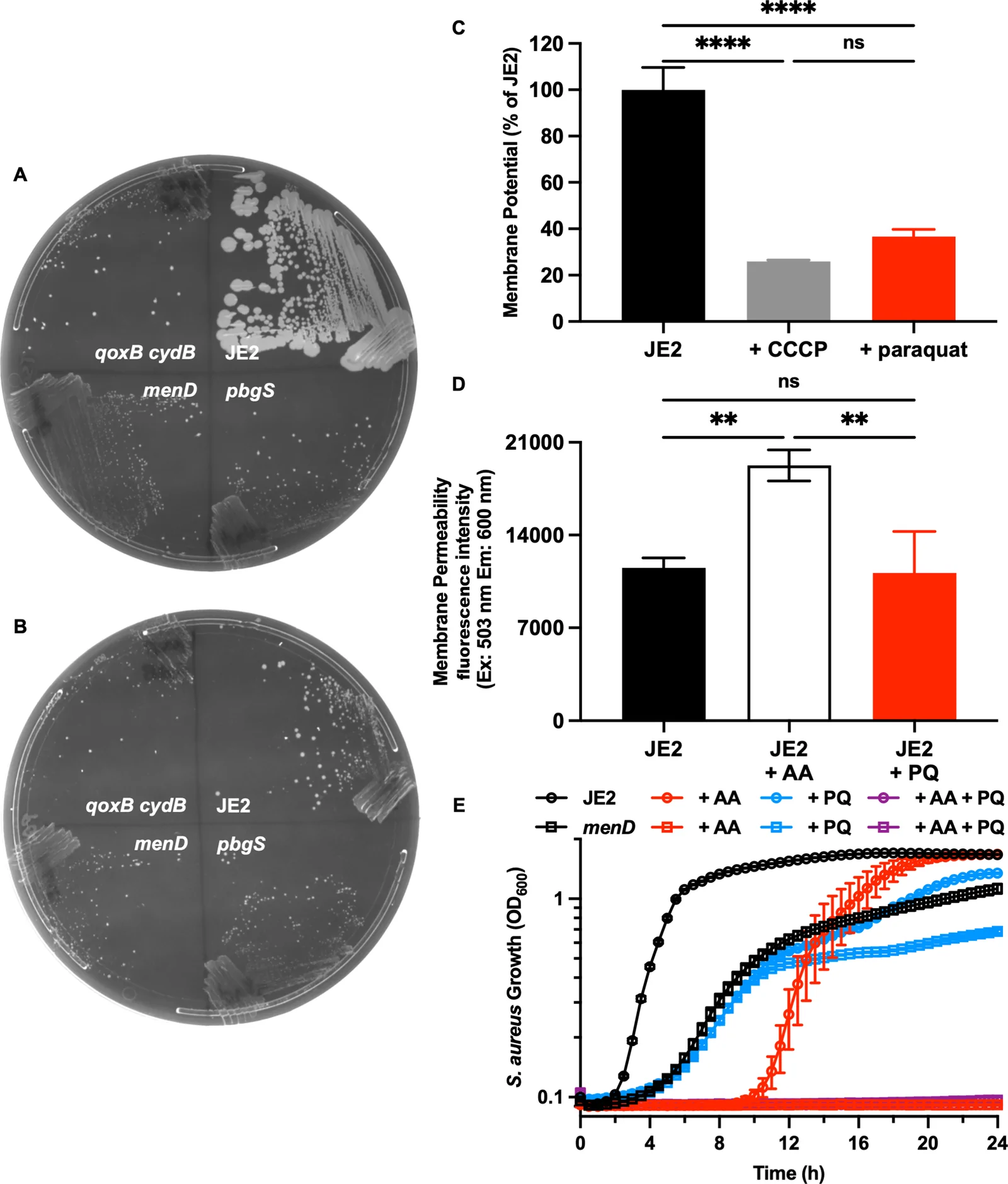
Paraquat induces a persister phenotype, sensitizing *S. aureus* to AA killing. (**A**) JE2, *menD*, *pbgS*, and *qoxB cydB* were streaked onto TSA to observe colony morphology. (**B**) JE2, *menD*, *pbgS*, and *qoxB cydB* were streaked onto TSA containing paraquat to observe colony morphology. (**C**) Membrane potential was assessed in JE2 treated with or without CCCP or paraquat. Fluorescence measurements (red, ex: 488 nm, em: 595 nm; green, ex: 488 nm, em: 530 nm) monitored the ratio of the dye inside and outside of the cell. Data are mean ± S.D. acquired in biological triplicate. Statistical significance was determined by one-way ANOVA, where ns = *P* > 0.05 and **** = *P* < 0.0001. (**D**) Membrane permeability was assessed in JE2 treated with or without AA or paraquat and ethidium bromide. Fluorescence measurements (ex: 503 nm, em: 600 nm) monitored the ability of ethidium bromide to enter the cell. Data are mean ± S.D. for data acquired in biological triplicate. Statistical significance was determined by one-way ANOVA, where ns = *P* > 0.05 and ** = *P* < 0.01. (**E**) JE2 or *menD* overnights were diluted and treated with or without AA and paraquat. Growth was monitored by optical density at 600 nm for 24 h. Data are mean ± S.E.M. for measurements acquired for six biological replicates per condition.

Previously, we reported that the oxidative stress generated by paraquat (42) exacerbates lipid peroxidation and thus exacerbates PUFA killing of *S. aureus* (19). Here, we add further mechanism to that finding by demonstrating that paraquat dose-dependently inhibits the growth of JE2 (Fig. S4A), but not the SCV strain *menD*, which is tolerant of millimolar concentrations of paraquat (Fig. S4B). To visually confirm the SCV resistance to paraquat, JE2, *menD*, *pbgS*, and *qoxB cydB* were streaked onto solid media with and without paraquat. On solid media, the SCV strains (*menD*, *pbgS*, and *qoxB cydB*) have much smaller colonies than JE2 (Fig. 5A). However, when the same strains are grown on solid medium containing paraquat, the strains all have similar small colony sizes (Fig. 5B), demonstrating that SCVs are resistant to the growth-inhibitory effects of paraquat observed for JE2. Further characterization of JE2 treated with paraquat demonstrates that paraquat ablates membrane potential to levels similarly observed with CCCP (Fig. 5C) and SCVs (Fig. 3B and Fig. 4A) without disrupting membrane permeability (Fig. 5D). Kinetic growth measurements show that 10 mM paraquat inhibits JE2 growth, causing *menD-*like growth, but does not alter *menD* growth (Fig. 5E). JE2 treated with AA has growth delayed to 10 h post-treatment, while *menD* treated with AA has no visible growth 24 h post-treatment, which is similar to both JE2 and *menD* treated with paraquat (Fig. 5E). These data demonstrate that paraquat ablates the membrane potential, resulting in a persister-like phenotype with metabolism similar to SCVs, and sensitizing *S. aureus* to AA killing.

### Cellular ATP levels predict AA killing of *S. aureus*

**Fig 6:**
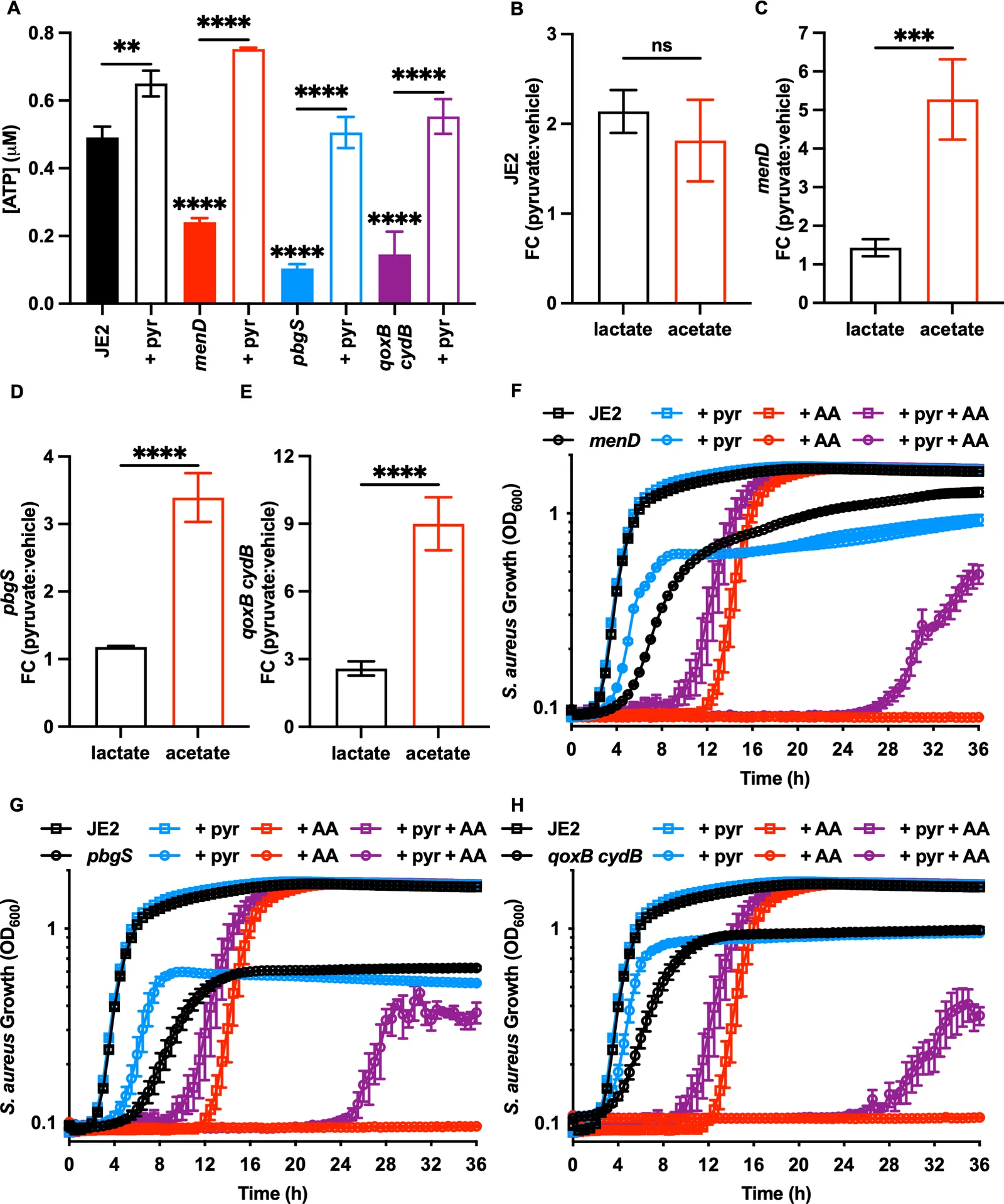
Sufficient intracellular ATP protects *S. aureus* from AA killing. (**A**) Cellular ATP levels were measured in JE2, *menD*, *pbgS*, and *qoxB cydB* grown in tryptic soy broth (TSB) with sodium chloride (vehicle) or sodium pyruvate (pyr) for 2.5 h. Data are mean ± S.D. acquired in biological triplicate. Statistical significance was determined by one-way ANOVA, where ** = *P* < 0.01 and **** = *P* < 0.0001. Comparisons are to untreated JE2 unless denoted in the graph. JE2 (**B**), *menD* (**C**), *pbgS* **(D**), or *qoxB cydB* (**E**) overnights were diluted and treated with or without vehicle or pyruvate and grown for 2 h. The media were derivatized and analyzed by LC-MS/MS for lactate and acetate levels. Data are mean ± S.D. of the fold change (FC) for the pyruvate:vehicle ratio for each analyte acquired in biological quadruplicate. Statistical significance was determined by *t*-test, where ns = *P* > 0.05, *** = *P* < 0.001, and **** = *P* < 0.0001. JE2, *menD* (**F**), *pbgS* (**G**), or *qoxB cydB* (**H**) overnights were diluted and treated with or without AA and pyruvate (pyr). Growth was monitored by optical density at 600 nm for 36 h. Data are mean ± S.E.M. for measurements acquired in biological quadruplicate.

The SCVs tested in this manuscript have defects in ATP production due to alterations to the ETC (Fig. 6A). Exogenous pyruvate is a source of ATP for SCVs due to the ability of *S. aureus* to convert pyruvate into ATP without altering the cellular NADH/NAD+ ratio (43). Supporting this, SCVs rapidly consume pyruvate, even when glucose is abundant, with ATP levels returning to baseline by 5 h post-pyruvate treatment, while not significantly altering ATP levels in JE2 (Fig. S5). When treated with exogenous pyruvate, intracellular ATP levels are increased in JE2, as well as the SCV strains (Fig. 6A), with a greater increase seen in the SCV strains. To better understand how pyruvate is being used by the cells, lactate and acetate were quantified in the media. The addition of pyruvate to the media increases both lactate and acetate in JE2 equally (Fig. 6B). However, in the SCV strains, acetate production increases significantly more than lactate production (Fig. 6C through E). These data indicate that the increased cellular ATP is through the conversion of pyruvate to ATP and acetate through the activity of pyruvate formate-lyase and phosphate acetyltransferase-acetyl kinase (44). Therefore, we tested the hypothesis that ATP deficiency sensitizes SCVs to AA killing. Exogenous pyruvate increases the growth rate of *menD* (Fig. 6F), *pbgS* (Fig. 6G), and *qoxB cydB* (Fig. 6H), but does not increase the growth rate of JE2. Exogenous pyruvate does increase the resistance of JE2 to AA treatment; however, it provides greater protection to *menD* (Fig. 6F), *pbgS* (Fig. 6G), and *qoxB cydB* (Fig. 6H). All SCV strains had no growth at 36 h post-AA treatment without pyruvate, but visible growth around 24 h when pyruvate was added to the media. These data implicate cellular ATP levels and therefore one or more cellular ATP-dependent processes as responsible for protecting *S. aureus* from PUFA killing.

### Nitrate respiration protects *S. aureus* from AA killing under anaerobic conditions

**Fig 7:**
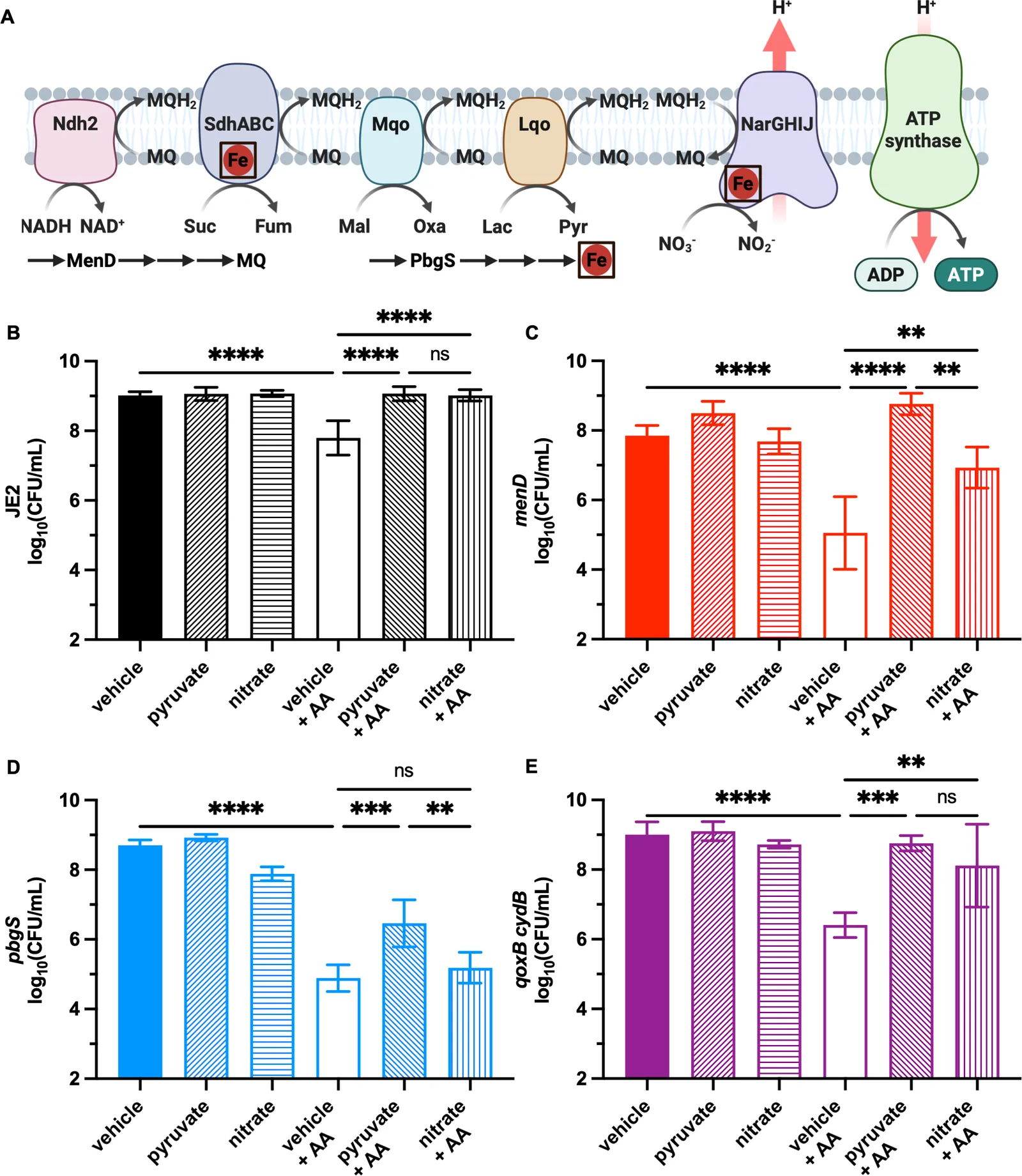
Anaerobic respiration protects *S. aureus* from AA killing when oxygen is limited. (**A**) The *S. aureus* anaerobic ETC with all mutated pathways studied in this manuscript annotated. JE2 (**B**), *menD* (**C**), *pbgS* (**D**), or *qoxB cydB* (**E**) were diluted and treated with and without AA and potassium sulfate (vehicle), potassium pyruvate, and potassium nitrate anaerobically. Each sample was incubated at 37°C for 16 h while turning the samples end-over-end. CFUs were enumerated by dilution plating onto solid medium. Data are mean ± S.D. acquired in biological quadruplicate. Statistical significance was determined by two-way ANOVA where ns = *P* > 0.05, ** = *P* < 0.01, *** = *P* < 0.001, and **** = *P* < 0.0001.

Oxygen is the preferred terminal electron acceptor of the *S. aureus* ETC (38). Under anaerobic conditions, *S. aureus* can express an alternative terminal oxidase complex, NarGHIJ, that uses nitrate instead of oxygen as the terminal electron acceptor (Fig. 7A) (45). So, we tested how oxygen concentration affects AA killing of *S. aureus* JE2 and SCVs. Using dilution plating, we determined that AA kills JE2 anaerobically and that the killing is completely reversed by the addition of exogenous pyruvate or nitrate (Fig. 7B). AA kills *menD* and the strain is completely protected by exogenous pyruvate (Fig. 7C). Exogenous nitrate provides incomplete protection against AA killing in *menD*, which is inconsistent with the necessity of menaquinol as the electron carrier to NarGHIJ for transfer to nitrate (Fig. 7C). Deletion of *pbgS* sensitizes *S. aureus* to AA anaerobically, which can be partially rescued by the exogenous addition of pyruvate (Fig. 7D). Nitrate does not protect *pbgS* from AA killing, consistent with NarGHIJ needing heme to transfer electrons to nitrate during anaerobic respiration (Fig. 7D). Finally, *qoxB cydB* is killed by AA and protected by both pyruvate and nitrate (Fig. 7E), consistent with these gene deletions only affecting the aerobic ETC. In total, these data indicate that ATP sufficiency is required for *S. aureus* to survive PUFA toxicity, regardless of oxygen concentration.

## DISCUSSION

PUFAs are abundant host fatty acids, not normally present in bacteria (18, 19). In response to cellular damage or invading pathogens, PUFAs are released in millimolar concentrations as a part of the inflammatory burst (18–20). Previously, we demonstrated that AA kills *S. aureus* through a lipid peroxidation mechanism where AA is oxidized to lipid electrophiles that form toxic protein PTMs (19). Here, we expand that discovery to other PUFAs and demonstrate that PUFA killing of *S. aureus* is not only mediated through lipid peroxidation, but that the autoxidation rate constants of PUFAs predict their ability to kill *S. aureus*. To our knowledge, no PUFA oxidases like the mammalian cyclooxygenases, lipoxygenases, or cytochrome P450s have been annotated in *S. aureus*, and consistent with this, our data demonstrate that lipid peroxidation in *S. aureus* is not mediated by enzymatic activity. We also previously demonstrated that the bactericidal lipid electrophiles are generated inside *S. aureus* (19). Using the exogenous lipid electrophile, 4-ONE, we discovered that exogenous lipid electrophiles are also toxic to *S. aureus*. These data show that lipid peroxidation is a broadly toxic process to *S. aureus* and that understanding the mechanisms of lipid peroxidation and electrophile detoxification in *S. aureus* will give insights into how we can more effectively help the mammalian immune system clear *S. aureus* infections.

We and others reported that unsaturated fatty acids increase the membrane permeability of *S. aureus* (19, 25–27). This phenomenon is also observed in mammalian cells and is due to the *cis* configuration of PUFA double bonds that prevent the fatty acids from packing tightly in the membrane (46). Here, we demonstrate that the membrane disruption by PUFAs correlates with the number of *cis* double bonds in each fatty acid. Other groups demonstrated that unsaturated fatty acids cause leaking of intracellular contents from *S. aureus* following treatment with unsaturated fatty acids (25–27). Capitalizing on this phenomenon, we described how PUFA membrane disruption resensitizes *S. aureus* to aminoglycoside antibiotics, even in strains with a low proton motive force (9). Consistent with this, we demonstrate that PUFAs dissipate the *S. aureus* membrane potential, correlative with the number of *cis* double bonds in the PUFA. This results in a decrease in cellular ATP and a smaller colony size than normally growing *S. aureus*. Bicarbonate rescues membrane potential and restores cellular ATP levels, consistent with previous reports (33, 34), even following AA treatment. Furthermore, bicarbonate protects *S. aureus* from AA killing, indicating that cellular ATP levels predict AA susceptibility. Decreased metabolic output is consistent with persister cell formation (8), which is generally associated with increased survival by *S. aureus* following toxic stressors (11–13). However, the data we present here demonstrate that cells exhibiting persister-like metabolism are more susceptible to killing by AA than respiring *S. aureus*.

*S. aureus* persister cells are metabolically dormant and typically arise in response to toxic stressors (8, 11–13). Due to the transient nature of metabolic phenotypes, genetically stable SCVs can be used to model persister cell metabolism (8, 14, 15). We used three different SCVs: *menD*, which is deficient in menaquinone biosynthesis (35); *pbgS*, which is deficient in heme biosynthesis (37); and *qoxB cydB*, which is deficient in both redundant aerobic terminal oxidases (38) to test how metabolism affects AA susceptibility. Both *menD* and *pbgS* are clinically relevant SCVs and comprise potential reservoirs for persistent *S. aureus* infections in patients (16, 36, 39). All three SCV strains tested here have decreased metabolism and membrane potential that sensitizes them to PUFA killing. Autoxidation rate constants for each PUFA predict the extent of killing in SCVs as well. The exogenous lipid electrophile, 4-ONE, kills the SCV strains better than JE2, indicating that metabolic protection of *S. aureus* following electrophile stress is a general proteotoxic stress response against both endogenous and exogenous electrophiles. The *menD* strain has significantly less AA taken into the cell, indicating even greater toxicity per molecule of AA. Metabolic alterations are considered a stress response that helps *S. aureus* survive in the mammalian host and form the basis of persistent *S. aureus* infections (15, 16). Here, we demonstrate that AA and PUFAs in general may be an effective therapeutic for eradicating metabolically dormant *S. aureus*.

We previously reported that paraquat, an herbicide that redox cycles in the membrane to generate oxidative stress (19, 42), exacerbates AA killing of *S. aureus* by providing oxidants to accelerate lipid peroxidation and the resulting lipid electrophile generation (19). Here, we amend that conclusion and show that paraquat treatment inhibits growth of JE2 dose-dependently until growth is similar to the SCV strain, *menD*, by ablating the membrane potential of JE2 without disrupting membrane integrity. Paraquat does not inhibit the growth of *menD* at the concentrations used to inhibit JE2 growth. Furthermore, on solid medium, paraquat induces SCV-like growth in JE2, but does not alter the colony size of the three SCV strains tested. This led to the conclusion that paraquat induces persister cell-like metabolism in *S. aureus*, and that this low metabolic state sensitizes *S. aureus* to AA killing. These data further demonstrate that lipid peroxidation is an effective antimicrobial strategy against metabolically dormant *S. aureus*.

*S. aureus* has diverse metabolic mechanisms to generate cellular energy, including aerobic respiration, anaerobic respiration, and glycolysis (7). This diverse metabolism is critical for *S. aureus* to cause infection in every niche of the mammalian host (3). Increasing cellular ATP levels is not as simple as adding more glucose due to carbon catabolite repression (47) and alterations of redox balance within the cell during glycolysis. During glycolysis, the NADH/NAD+ ratio is skewed toward NADH (45). When the ETC is functional, the type-2 NADH:menaquinone oxidoreductases convert NADH to NAD+, restoring the redox balance of the cell (48). Without a functional ETC, redox balance is restored in SCVs and other non-respiring strains by converting pyruvate to lactate, followed by excretion from the cell (44). Glycolytic *S. aureus* can convert pyruvate to ATP through acetyl-CoA without disrupting the cellular redox balance (43). Emphasizing how important pyruvate is as an energy source, *S. aureus* expresses four pyruvate transporters (6). Adding pyruvate to the media increases cellular ATP in JE2 and the SCV strains tested, with a greater increase coming in the SCV strains. Exogenous pyruvate provides a small improvement in AA susceptibility to JE2 while not improving basal growth, likely due to JE2 running oxidative phosphorylation and being fully ATP replete. Exogenous pyruvate increases the growth rate for all SCV strains and protects them from AA killing. We previously reported data that implies *S. aureus* SCVs consume exogenous pyruvate by 3 h post-treatment (34). Consistent with that finding, we show here that ATP levels return to baseline in *menD* by 5 h post-pyruvate treatment, while they remain stable in JE2 across time, leading us to speculate that sustained pyruvate consumption without disruption to cellular redox balance will provide wild-type level protection in SCVs. These data indicate that AA susceptibility is modulated through metabolism and that increasing cellular ATP levels protects metabolically dormant *S. aureus* from AA killing.

*S. aureus* is the leading cause of infective osteomyelitis (49). Bone is oxygen-deplete (50), presenting another host niche where metabolic flexibility is critical for *S. aureus* infection. When oxygen levels are depleted, *S. aureus* generates energy through glycolysis, unless excess nitrate is present. In this case, *S. aureus* can generate energy through anaerobic respiration, where the NarGHIJ complex uses nitrate as a terminal electron acceptor, restoring ETC function (45). AA kills all of the strains (JE2, *menD*, *pbgS*, and *qoxB cydB*) tested anaerobically better than aerobically, and all of the strains are protected by exogenous pyruvate. Exogenous nitrate provides complete anaerobic protection from AA killing in both JE2 and *qoxB cydB*, consistent with both strains being able to express a fully active NarGHIJ to use nitrate as a terminal electron acceptor. Nitrate partially protects *menD*, which is inconsistent with an inactive ETC and a finding we currently do not understand. Nitrate does not protect *pbgS*, which is consistent with NarGHIJ needing heme to transfer electrons from menaquinol to nitrate as the final step of the ETC. While we do not know the identity of the oxidants that initiate lipid peroxidation anaerobically, these data demonstrate that AA kills these strains anaerobically. Together, these data demonstrate that lipid peroxidation of PUFAs effectively kills *S. aureus* even when oxygen is depleted.

**Fig 8:**
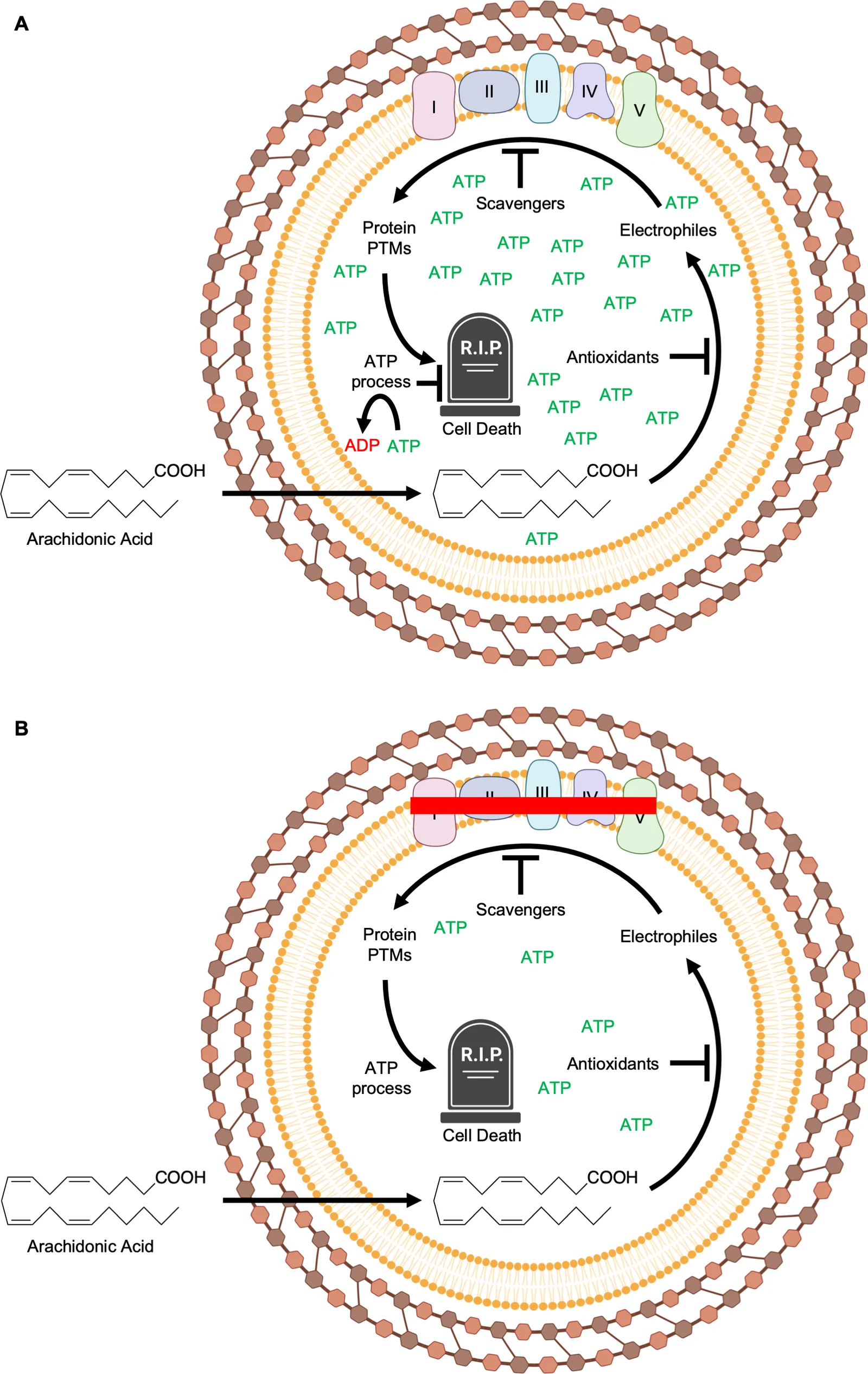
The *S. aureus* ETC modulates susceptibility to PUFAs by maintaining ATP sufficiency. PUFAs enter the *S. aureus* cell where they are oxidized to lipid electrophiles, which can form protein post-translational modifications. (**A**) When the ETC is functional or an alternative carbon source like pyruvate is supplied, there is sufficient cellular ATP to fuel the ATP-dependent processes that protect *S. aureus* from lipid electrophile-mediated killing. (**B**) However, when the ETC is not functional, such as in an anaerobic environment or in SCVs and persister cells, ATP levels are insufficient to fuel these processes and the ability of *S. aureus* to survive PUFA killing is diminished.

Glycolytic *S. aureus*, whether an SCV or persister cell, has long been a target for therapeutic intervention in treating recurrent *S. aureus* infections (15). The altered metabolism in these cells makes them resistant to antibiotics (9, 10), oxidative stressors (11), and the host immune system (12, 13), and thus a reservoir for persistent *S. aureus* infections (16, 36, 39). PUFAs kill both respiring and glycolytic *S. aureus* through a lipid peroxidation mechanism, with a stronger phenotype against glycolytic cells. We report an inverse correlation between PUFA killing and cellular ATP levels and were able to directly show that increasing cellular ATP is protective against PUFA killing. This implies that ATP-dependent processes are essential for *S. aureus* to survive PUFA-induced proteotoxicity (Fig. 8). ATP fuels nearly all cellular processes, meaning that there are likely multiple ATP-dependent processes that protect *S. aureus* from PUFA toxicity. We previously demonstrated that lipid electrophiles modify proteins across much of the *S. aureus* proteome, implicating that multiple cellular processes may be altered through PUFA toxicity and necessitating a multipronged defense using ATP-dependent pathways. Current and future studies in the group to understand the role of cellular ATP in PUFA defense include investigating cell wall biosynthesis and maintenance to provide a protective barrier against PUFA entry into cells; efflux pumps that export PUFAs before they are esterified into the membrane and oxidized into antimicrobial lipid electrophiles; proteolytic systems that degrade electrophile-damaged proteins before they accumulate and cause cell death; and protein synthesis that rapidly replaces deleteriously damaged essential proteins. In total, the data presented in this manuscript demonstrate that PUFAs are potent, host-derived antimicrobial compounds with the ability to kill *S. aureus* regardless of the metabolic machinery being used to generate energy. Further defining the mechanisms of PUFA killing and resistance in *S. aureus* will identify and validate pathways that can be targeted by future antimicrobial compounds to sensitize *S. aureus* to lipid peroxidation and killing by the mammalian host.

## MATERIALS AND METHODS

### Materials

All bacterial strains used in this manuscript are listed and described in Table 1. Micropipette tips, serological tips, aeration culture tubes, aeration culture lids, ethidium bromide, menadione, sodium hydroxide, ethyl acetate, paraquat hydrochloride, sodium chloride, potassium sulfate, acetone, and potassium nitrate were purchased from VWR International (Radnor, PA). Micropipettes, pipette controllers, MS vials, MS caps, MS inserts, potassium pyruvate, sodium bicarbonate, and the BCA kit were purchased from Fisher Scientific (Waltham, MA). 4-Oxo-2-nonenal, palmitic acid, oleic acid, linoleic acid, docosahexaenoic acid, arachidonic acid-d_8_, and CCCP were purchased from Cayman Chemical (Ann Arbor, MI). Arachidonic acid and α-tocopherol were purchased from TCI America (Portland, OR). 96-well round-bottom plates, 96-well clear-bottom black-walled plates, 100 mm plates, 150 mm plates, 15 mL conical tubes, and 50 mL conical tubes were purchased from Corning Incorporated (Corning, NY). Sodium pyruvate was purchased from BeanTown Chemicals (Hudson, NH). Hemin chloride was purchased from MilliporeSigma (Burlington, MA). Lysostaphin was purchased from BioVendor (Asheville, NC). Argon gas, nitrogen gas, and 95:5 nitrogen:hydrogen gas mix were purchased from Airgas (Radnor, PA). Microcentrifuge tubes were purchased from USA Scientific (Ocala, FL). Tryptic soy agar (TSA) and tryptic soy broth (TSB) were purchased from BD Difco (Franklin Lakes, NJ). All model figures were created using BioRender (Toronto, Ontario, Canada). All chemical structures were made in ChemDraw 22.2.0 (Revvity Signals Software, Waltham, MA). All data were plotted and statistical analyses were performed in Prism 10 (GraphPad, La Jolla, CA).

**TABLE 1:** Bacterial strains used in this manuscript

Strain and genotype	Manuscript name	Description	Source
*S. aureus* USA300 JE2	JE2	Wild-type USA3000 community-acquired methicillin-resistant *S. aureus* isolate	(51)
*S. aureus* USA300 JE2 *ΔmenD*	*menD*	In-frame, unmarked deletion of *menD* (*SAUSA300_0946*)	(34)
*S. aureus* USA300 JE2 *pbgS::Tn*	*pbgS*	NTML transposon insertion strain NE1845 (*SAUSA300_1615*)	(51)
*S. aureus* USA300 JE2 *qoxB::Tn ΔcydB*	*qoxB cydB*	NTML transposon insertion strain NE732 (*SAUSA300_0962*) with an in-frame, unmarked deletion of *cydB* (*SAUSA300_0987*)	(34)
*S. aureus* USA300 JE2, empty vector	JE2, vec	Wild-type USA3000 community-acquired methicillin-resistant *S. aureus* isolate containing the pOS1.P*_lgt_* empty vector	(34)
*S. aureus* USA300 JE2, *cydB* complementation	JE2, *cydB*	Wild-type USA3000 community-acquired methicillin-resistant *S. aureus* isolate containing the pOS1.P*_lgt_.cydB* complementation vector constitutively expressing *cydB*	(34)
*S. aureus* USA300 JE2 *qoxB::Tn ΔcydB*, empty vector	*qoxB cydB*, vec	NTML transposon insertion strain NE732 (*SAUSA300_0962*) with an in-frame, unmarked deletion of *cydB* (*SAUSA300_0987*) containing the pOS1.P*_lgt_* empty vector	(34)
*S. aureus* USA300 JE2 *qoxB::Tn ΔcydB*, *cydB* complementation	*qoxB cydB*, *cydB*	NTML transposon insertion strain NE732 (*SAUSA300_0962*) with an in-frame, unmarked deletion of *cydB* (*SAUSA300_0987*) containing the pOS1.P*_lgt_.cydB* complementation vector constitutively expressing *cydB*	(34)

### *S. aureus* bacterial cultures

Unless otherwise noted, all *S. aureus* strains were streaked to TSA and grown for 24 h at 37°C. SCV strains were grown for 48 h on TSA. Strains harboring the pOS1 complementation plasmid were streaked to TSA containing 10 μg/mL chloramphenicol and grown as above. Single bacterial colonies were inoculated into aeration culture tubes with aeration culture lids containing TSB ± 10 µg/mL chloramphenicol to create bacterial overnights. Aeration culture tubes were placed at a 45° angle and grown at 37°C with orbital shaking at 180 rpm for 16 h in an Innova44 shaking incubator (Eppendorf, Hauppauge, NY).

### *S. aureus* kinetic growth curves

All kinetic growth curve experiments were performed on a BioTek Epoch 2 plate reader (Agilent, Santa Clara, CA). The plate reader was set to 37°C, and *S. aureus* cells were kept suspended with linear shaking at 567 cpm (3 mm excursion) between reads (19). Optical density at 600 nm (OD_600_) readings were taken every 30 min for 24, 36, or 48 h, depending on the experiment.

### Kinetic growth curve following fatty acid treatment

JE2 or *menD* overnights were diluted 1,000-fold (10^6^ CFU/mL starting) into 100 μL of TSB containing 0, 50, or 100 μM OA, LA, AA, or DHA in a 96-well round-bottom plate. JE2 was treated with 100 μM fatty acid, while *menD* was treated with 50 μM fatty acid due to the increased sensitivity to PUFA killing in the SCV strains. Growth was monitored for six biological replicates for 48 h per condition, as described above.

### CFU enumeration of JE2 following fatty acid treatment

JE2 overnights were diluted 1,000-fold (10^6^ CFU/mL starting) into 100 μL of TSB containing 0 or 100 μM OA, LA, AA, or DHA, and 0 or 50 μM αTOH in a 96-well round-bottom plate. The plates were grown at 37°C with orbital shaking at 180 rpm for 4 h in a shaking incubator. Following incubation, each well was diluted 10^0^ through 10^−7^ in phosphate-buffered saline (PBS). Ten microliters of each dilution was spot plated onto TSA and incubated overnight at 37°C, followed by CFU enumeration.

### 4-ONE growth curves

JE2 and *menD* overnights were diluted 1,000-fold (10^6^ CFU/mL starting), and *pbgS* and *qoxB cydB* overnights were diluted 100-fold (10^6^ CFU/mL starting) into 100 μL of 1:1 TSB:PBS containing 0, 16, 32, 64, 128, or 256 μM 4-ONE in a 96-well round-bottom plate. Growth was monitored for three biological replicates for 24 h per condition as described above.

### *S. aureus* JE2 membrane permeability following fatty acid treatment

JE2 overnights were pelleted by centrifugation, washed twice in PBS, and suspended in PBS equal to the original media volume. Twenty microliters of each bacterial replicate was combined with 80 μL of 0 or 50 μM PA, LA, AA, or DHA in a 96-well clear-bottom black-walled plate. Ethidium bromide was added to each well to a final concentration of 2 ng/μL, and intercalation into genomic DNA was measured kinetically on a BioTek Cytation 5 (Agilent) (52). The plate was incubated at 37°C with linear shaking at 567 cpm (3 mm excursion) between reads. Fluorescence intensity was measured every 30 min at the excitation and emission wavelengths of 503 and 600 nm, respectively.

### *S. aureus* JE2 membrane potential following fatty acid treatment

JE2 overnights were pelleted by centrifugation and resuspended in PBS. The OD_600_ of each replicate was adjusted to OD_600_ = 0.5 with PBS. One milliliter of cells was combined with 0 or 10 μM CCCP, PA, LA, AA, or DHA and incubated at room temperature for 10 min. Five microliters of MycoStain It Green dye from the MycoLight Ratiometric Bacterial Membrane Potential Kit (AAT Bioquest, Pleasanton, CA) and 200 μL of each replicate were transferred to a 96-well clear-bottom black-walled plate. Membrane potential was monitored at excitation/emission wavelengths of 488/595 nm (red) and 488/530 nm (green) on a Cytation 5 plate reader every 10 min for 1 h at 25°C with linear shaking at 567 cpm (3 mm excursion) between reads. MycoStain It Green dye fluoresces green extracellularly and red intracellularly, making the red:green ratio a measure of membrane potential.

### Intracellular ATP levels following AA treatment

JE2 overnights were pelleted by centrifugation and resuspended in PBS. The OD_600_ of each replicate was adjusted to OD_600_ = 0.1 with PBS. One milliliter of cells was combined with vehicle (5 μL of ethanol), 10 μM CCCP, or 50 μM AA and incubated for 1 h at room temperature. One hundred microliters of each replicate was transferred to a 96-well clear-bottom black-walled plate and combined with 100 μL of CellTiter-Glo 2.0 reagent (Promega, Madison, WI). Total luminescence was read from 300 to 700 nm at room temperature on a Cytation 5 plate reader after linear shaking for 2 min at 567 cpm (3 mm excursion) and a 10 min pause to stabilize the luminescence signal. For each experiment, an ATP standard curve was generated using concentrations of ATP ranging from 1 to 0.0078 μM to calculate the ATP concentration in each sample.

### *S. aureus* growth on solid medium with arachidonic acid

TSA plates were poured with 0 or 200 μM AA. JE2 overnights were diluted 100,000-fold in PBS, and 100 μL (1,000 CFU) was spread on each plate. The plates were incubated overnight at 37°C before being imaged on an Azure Biosystems 600 Imager (Dublin, CA).

### Membrane potential following AA and bicarbonate treatment

JE2 overnights were pelleted by centrifugation and resuspended in PBS. The OD_600_ of each replicate was adjusted to OD_600_ = 1.0 with PBS. A volume of 250 μL of cells was combined with 0 or 10 μM CCCP, 0 or 25 μM AA, and 25 mM sodium chloride (vehicle) or 25 mM sodium bicarbonate and incubated at room temperature for 10 min. The membrane potential was measured as described above.

### Intracellular ATP levels following AA and bicarbonate treatment

JE2 overnights were pelleted by centrifugation and resuspended in PBS. The OD_600_ of each replicate was adjusted to OD_600_ = 0.1 with PBS. One milliliter of cells was combined with 0 or 10 μM CCCP, 0 or 25 μM AA, and 25 mM sodium chloride (vehicle) or 25 mM sodium bicarbonate and incubated for 1.5 h at room temperature. The ATP concentration of each sample was measured as described above.

### CFU enumeration following AA and bicarbonate treatment

JE2 overnights were diluted 1,000-fold (10^6^ CFU/mL starting) into 100 μL of TSB + 100 mM HEPES pH 7.3 (34) containing 0 or 100 μM AA and 25 mM sodium chloride (vehicle) or 25 mM sodium bicarbonate in a 96-well round-bottom plate. The plates were grown in an Epoch 2 plate reader at 37°C for 17 h, and *S. aureus* cells were kept suspended with linear shaking at 567 cpm (3 mm excursion). Following incubation, each well was diluted 10^0^ through 10^−7^ in PBS. Ten microliters of each dilution was spot-plated onto TSA and incubated overnight at 37°C, followed by CFU enumeration.

### *S. aureus menD* complemented membrane potential following AA treatment

JE2 and *menD* overnights were prepared as described above, with the exception that *menD* was complemented with the addition of 5 μM menadione to the media. Overnights were diluted 100-fold into TSB with and without 5 μM menadione and grown for 4 h at 37°C under the same conditions as the overnight growth. The outgrowths were pelleted by centrifugation and resuspended in PBS. The OD_600_ of each replicate was adjusted to OD_600_ = 0.5 with PBS. One milliliter of cells was combined with 0 or 10 μM CCCP and 0 or 10 μM AA and incubated at room temperature for 10 min. The membrane potential was measured as described above.

### Intracellular ATP levels in *menD*

JE2 and *menD* overnights were diluted 100-fold into TSB and grown for 4 h at 37°C under the same conditions as the overnight growth. The outgrowths were pelleted by centrifugation and resuspended in PBS. The OD_600_ of each replicate was adjusted to OD_600_ = 0.1 with PBS. One milliliter of cells was combined with or without 10 μM CCCP and incubated for 1 h at room temperature. The ATP concentration of each sample was measured as described above.

### CFU enumeration of complemented *menD* treated with AA treatment

JE2 and *menD* overnights were prepared as described above. JE2 and *menD* overnights grown with and without 5 μM menadione were diluted 100-fold (10^7^ CFU/mL starting) into 100 μL of TSB containing 0 or 5 μM menadione, 0 or 100 μM AA, and 0 or 50 μM α-tocopherol in a 96-well round-bottom plate. The plates were grown at 37°C with orbital shaking at 180 rpm for 4 h in a shaking incubator. Following incubation, each well was diluted 10^0^ through 10^−7^ in PBS. Ten microliters of each dilution was spot-plated onto TSA and incubated overnight at 37°C, followed by CFU enumeration.

### Fatty acid extraction from *S. aureus*

JE2 and *menD* overnights were diluted fivefold into TSB with and without 100 μM AA. The samples were grown at 37°C with orbital shaking at 180 rpm for 0, 30, 60, or 120 min in a shaking incubator. Cells were pelleted by centrifugation and washed twice with PBS. The cell wall was degraded for 30 min at 37°C in 300 μL of 10 mM MgSO_4_ in PBS containing 20 μg of lysostaphin and 10 nanomoles of arachidonic acid-d_8_ (AA-d_8_) as an internal standard. Lipids were extracted from the samples with 300 μL of methanol and 600 μL of methyl tert-butyl ether. Centrifugation was used to facilitate phase extraction, and the organic layer (top) was transferred to clean glass tubes. The aqueous layer was extracted once more with 600 μL of methyl tert-butyl ether. Following centrifugation, the organic layers were combined and dried to completion under a stream of nitrogen gas in a nitrogen evaporator (Organomation, Berlin, MA). Esterified fatty acids were hydrolyzed from phospholipid pools by incubating each sample under nitrogen gas for 1 h at 37°C in 1 mL of 1 N NaOH prepared in methanol before drying the samples to completion in a nitrogen evaporator. Samples were dissolved in 150 μL of 2:1 methanol:0.1% formic acid for LC-MS/MS analysis.

Protein in the aqueous layer was precipitated with a 4× volume of ice-cold acetone, and a protein pellet was formed by centrifugation at maximum speed for 10 min at 4°C. Proteins were solubilized in 1% sodium dodecyl sulfate in PBS, facilitated by probe sonication (Fisher Scientific) and boiling. Proteins were quantified as a biomass normalization factor using a BCA kit (Pierce, Fisher Scientific).

### LC-MS/MS analysis of AA in *S. aureus*

The extracted and base-hydrolyzed samples were analyzed at the LSU School of Veterinary Medicine Mass Spectrometry Resource Center on a Shimadzu 8060NX triple quadrupole mass spectrometer interfaced with a Shimadzu Nexera XS 40 series UPLC and a Shimadzu CTO-40S column oven (Shimadzu Scientific Instruments, Columbia, MD). Ten microliters of each sample or blank was analyzed in negative ionization mode. Chromatographic separation was achieved using a Phenomenex Kinetex C_18_ (50 mm length, 2.1 mm internal diameter, 1.7 μm particle size, 100 Å pore size) column with a Phenomenex Security Guard C_18_ (2.1 mm internal diameter) guard column (Phenomenex, Torrance, CA). Mobile phase A was 0.1% formic acid in water, and mobile phase B was 0.1% formic acid in acetonitrile. The column oven was set to 25°C, and the flow rate was set to 0.4 mL/min for the entire analysis. The starting condition was 20% B, which was held for 2 min. The B mobile phase was then ramped to 98% over the next 3 min. The column was washed at 98% B for 3 min, then equilibrated to 20% B for 2 min. All instrument voltages were determined and optimized empirically prior to the analysis. The fatty acid *m/z* transitions were 303.2 to 259.2 for AA and 311.2 to 267.2 for AA-d_8_. Quantification was achieved for each sample by comparing the AUC of AA to the AUC of AA-d_8_. Each sample was normalized to total protein to account for differences in starting material.

### *S. aureus pbgS* complemented membrane potential following AA treatment

JE2 and *pbgS* overnights were prepared as described above, with the exception that *pbgS* was complemented with the addition of 5 μM hemin chloride to the media. JE2 overnights were diluted 100-fold into TSB, and *pbgS* overnights were diluted 10-fold into TSB with and without 5 μM hemin chloride, and grown for 4 h at 37°C under the same conditions as the overnight growth. The *pbgS* strain grows to a lower confluence than JE2, so the dilution difference was necessary to make sure both strains started at 10^7^ CFU/mL. The outgrowths were pelleted by centrifugation and resuspended in PBS. The OD_600_ of each replicate was adjusted to OD_600_ = 0.5 with PBS. One milliliter of JE2 cells was combined with 0 or 10 μM CCCP, and 1 mL of *pbgS* cells was combined with 0 or 10 μM CCCP, and 0 or 10 μM AA, and incubated at room temperature for 10 min. The membrane potential was measured as described above.

### CFU enumeration of complemented *pbgS* with AA treatment

JE2 and *pbgS* overnights grown with and without 5 μM hemin chloride were diluted 100-fold or 10-fold (10^7^ CFU/mL starting), respectively, into 100 μL of TSB containing 0 or 5 μM hemin chloride, 0 or 100 μM AA, and 0 or 50 μM α-tocopherol in a 96-well round-bottom plate. The plates were grown at 37°C with orbital shaking at 180 rpm for 4 h in a shaking incubator. Following incubation, each well was diluted 10^0^ through 10^−7^ in PBS. Ten microliters of each dilution was spot plated onto TSA and incubated overnight at 37°C, followed by CFU enumeration.

### Kinetic growth curve of complemented *qoxB cydB* with AA treatment

JE2 and *qoxB cydB* containing pOS1.P*_lgt_* (vec = empty vector) or pOS1.P*_lgt_.cydB* (*cydB* = complementation vector) were grown in TSB containing 10 μg/mL chloramphenicol for the entire experiment to maintain the plasmids. JE2 and *qoxB cydB* overnights were diluted 1,000-fold or 100-fold (10^6^ CFU/mL starting), respectively, into 100 μL of TSB containing 0 or 100 μM AA in a 96-well round-bottom plate. Growth was monitored for six biological replicates for 48 h per condition as described above.

### CFU enumeration of antioxidant-treated *qoxB cydB* with AA treatment

The *qoxB cydB* overnights were diluted 100-fold (10^6^ CFU/mL starting) into 100 μL of TSB containing 0 or 100 μM AA and 0 or 50 μM α-tocopherol in a 96-well round-bottom plate. The plates were grown at 37°C with orbital shaking at 180 rpm for 4 h in a shaking incubator. Following incubation, each well was diluted 10^0^ through 10^−7^ in PBS. Ten microliters of each dilution was spot plated onto TSA and incubated overnight at 37°C, followed by CFU enumeration.

### Bacterial growth on paraquat-embedded solid medium

TSA plates were poured with 0 or 25 mM paraquat. JE2, *menD*, *pbgS*, and *qoxB cydB* were streaked from glycerol stocks onto the solid medium. The plates were incubated for 24 h at 37°C before being imaged on an Azure Biosystems 600 Imager (Dublin, CA).

### Kinetic growth curve with paraquat treatment

JE2 or *menD* overnights were diluted 1,000-fold (10^6^ CFU/mL starting) into 100 μL of TSB containing 12.8, 6.4, 3.2, 1.6, 0.8, 0.4, or 0 mM paraquat in a 96-well round-bottom plate. Growth was monitored for six biological replicates for 24 h per condition as described above.

### *S. aureus* JE2 membrane potential following paraquat treatment

JE2 overnights were pelleted by centrifugation and resuspended in PBS. The OD_600_ of each replicate was adjusted to OD_600_ = 0.75 with PBS. One milliliter of JE2 cells was combined with 0 or 10 μM CCCP and 0 or 100 mM paraquat and incubated at room temperature for 1 h. The membrane potential was measured as described above.

### *S. aureus* JE2 membrane permeability following paraquat treatment

JE2 overnights were pelleted by centrifugation, washed twice in PBS, and suspended in PBS equal to the original media volume. Twenty microliters of each bacterial replicate was combined with 80 μL of 0 or 50 μM AA and 0 or 10 mM paraquat in a 96-well clear-bottom black-walled plate. Ethidium bromide was added to each well to a final concentration of 2 ng/μL, and intercalation into genomic DNA was measured as described above.

### Kinetic growth curve following paraquat and AA treatment

JE2 or *menD* overnights were diluted 1,000-fold (10^6^ CFU/mL starting) into 100 μL of TSB containing 0 or 10 mM paraquat and 0 or 50 μM AA in a 96-well round-bottom plate. Growth was monitored for six biological replicates for 24 h per condition as described above.

### Intracellular ATP levels with and without pyruvate treatment

Overnights were diluted 100-fold for JE2 and *menD* or 10-fold for *pbgS* and *qoxB cydB* into TSB with 20 mM sodium chloride (vehicle) or 20 mM sodium pyruvate, and grown for 2 h at 37°C under the same conditions as the overnight growth. The outgrowths were pelleted by centrifugation and resuspended in PBS. The OD_600_ of each replicate was adjusted to OD_600_ = 0.1 with PBS. The ATP concentration of each sample was measured as described above.

### LC-MS/MS quantification of lactate and acetate in the media with and without pyruvate treatment

Overnights were diluted 100-fold (10^7^ CFU/mL starting) for JE2 and *menD* or 10-fold (10^7^ CFU/mL starting) for *pbgS* and *qoxB cydB* into TSB with 25 mM potassium sulfate (vehicle) or 50 mM potassium pyruvate and grown for 2 h at 37°C under the same conditions as the overnight growth. The outgrowths were pelleted by centrifugation, and 200 μL of media was combined with 1.8 mL of pyridine reaction buffer (21.6 mL of 6 N HCl, 17.2 mL of pyridine, and 161.2 mL of water, pH 5.0), 50 μL of 1 M 1-ethyl-3-(3-dimethylaminopropyl)carbodiimide (Fisher Scientific), and 50 μL of 1 M O-benzylhydroxylamine (TCI America) to derivatize as previously described (34, 53). The derivatization was allowed to proceed at room temperature for 1 h before being extracted thrice with 3 mL of ethyl acetate. Phase separation was facilitated by centrifugation following each extraction, and the organic layers were combined and dried to completion under a stream of nitrogen gas. Each sample was dissolved in 2:1 methanol:water for LC-MS/MS analysis.

The samples were analyzed at the LSU School of Veterinary Medicine Mass Spectrometry Resource Center on a Shimadzu 8060NX triple quadrupole mass spectrometer interfaced with a Shimadzu Nexera XS 40 series UPLC and a Shimadzu CTO-40S column oven. Two microliters of each sample, standard, or blank was analyzed in positive ionization mode. Chromatographic separation was achieved using a Phenomenex Kinetex C_8_ (150 mm length, 2.1 mm internal diameter, 2.6 μm particle size, 100 Å pore size) column with a Phenomenex Security Guard C_8_ (2 mm internal diameter) guard column. Mobile phase A was 0.1% formic acid in water, and mobile phase B was 0.1% formic acid in acetonitrile. The column oven was set to 25°C, and the flow rate was set to 0.4 mL/min for the entire analysis. The starting condition was 10% B, which was held for 2 min. Mobile phase B was then ramped to 50% over the next 23 min. The column was washed at 98% B for 10 min, then equilibrated to 10% B for 5 min. All instrument voltages were determined and optimized empirically prior to the analysis. The *m/z* transitions of 166.1 Da to 65.1 Da and 196.1 Da to 65.1 Da were used to monitor acetate and lactate, respectively. Quantification was achieved using an external calibration line of derivatized acetate and lactate.

### Kinetic growth curve following pyruvate and AA treatment

JE2 and *menD* overnights were diluted 1,000-fold, and the *pbgS* and *qoxB cydB* overnights were diluted 100-fold (10^6^ CFU/mL starting) into 100 μL of TSB containing 20 mM sodium chloride (vehicle), 20 mM sodium pyruvate, and 0 or 50 μM AA in a 96-well round-bottom plate. Growth was monitored for four biological replicates for 36 h per condition as described above.

### Intracellular ATP levels with and without pyruvate treatment across time

JE2 and *menD* overnights were diluted 100-fold into TSB with 20 mM sodium chloride (vehicle) or 20 mM sodium pyruvate and grown for 2.5 or 5 h at 37°C under the same conditions as the overnight growth. The outgrowths were pelleted by centrifugation and resuspended in PBS. The OD_600_ of each replicate was adjusted to OD_600_ = 0.1 with PBS. The ATP concentration of each sample was measured as described above.

### Anaerobic CFU enumeration following treatment with AA, pyruvate, and nitrate

JE2, *menD*, *pbgS*, and *qoxB cydB* were streaked to TSA and grown at 37°C for 48 h in an anaerobic chamber (Coy Laboratory Products, Grass Lake, MI). All subsequent steps were performed in an anaerobic chamber kept below 10 ppm oxygen. Single colonies were inoculated into 1 mL of TSB (equilibrated to anaerobic conditions for at least 1 week) in 1.5 mL tubes and incubated for 24 h at 37°C while turning end-over-end to keep the bacteria suspended. The JE2 and *menD* overnights were diluted 100-fold, and the *pbgS* and *qoxB cydB* overnights were diluted 10-fold (10^7^ CFU/mL starting) into 500 μL of TSB containing 25 mM potassium sulfate (vehicle), 0 or 50 mM potassium pyruvate, 0 or 50 mM potassium nitrate, and 0 or 50 μM AA in 1.5 mL tubes. The tubes were incubated for 16 h at 37°C while turning end-over-end to keep the bacteria suspended. Following incubation, each sample was diluted 10^0^ through 10^−7^ in PBS. Ten microliters of each dilution was spot plated onto TSA and incubated overnight at 37°C, followed by CFU enumeration.

## Data Availability

All data in this manuscript and reagents that are not commercially available are available upon request from the corresponding author.

## SUPPLEMENTAL MATERIAL

Supplemental figures jb.00249-26-s0001.docx Fig. S1 to S5.

Table S1 jb.00249-26-s0002.xlsx AA uptake in *S. aureus* LC-MS/MS data.

ASM does not own the copyrights to Supplemental Material that may be linked to, or accessed through, an article. The authors have granted ASM a non-exclusive, world-wide license to publish the Supplemental Material files. Please contact the corresponding author directly for reuse.
