# Supplementary material for: Polyunsaturated fatty acids: host-derived antimicrobials against glycolytic Staphylococcus aureus
Source: J Bacteriol. 2026 Sep 17;208(10):e00249-26. doi: 10.1128/jb.00249-26 (PMC13689550; doi:10.1128/jb.00249-26)
Supplement: Supplementary file 1 [file jb.00249-26-s0001.docx]

**Polyunsaturated fatty acids: host-derived antimicrobials against glycolytic *Staphylococcus aureus***

Alex R. Stackhouse^a^, Asif Iqbal^a^, D. Grace Ross^a^, Wyatt W. Wittliff^a,b^, Kirsten E. Rico^a^, Bennett J. Blank^a^, Brooke M. LeBreton^a^, Sara P. Bucher^a^, Michelle Y. MacIntyre^a^, William N. Beavers^a,b,#^

^a^Department of Pathobiological Sciences, Louisiana State University and Agricultural and Mechanical College, School of Veterinary Medicine, Baton Rouge, Louisiana, USA

^b^Louisiana State University School of Veterinary Medicine Mass Spectrometry Resource Center, Louisiana State University and Agricultural and Mechanical College, School of Veterinary Medicine, Baton Rouge, Louisiana, USA

Alex R. Stackhouse and Asif Iqbal contributed equally to this work. Author order was determined by seniority in the laboratory.

^#^Address correspondence to:

William N. Beavers, Ph.D.

LSU School of Veterinary Medicine

Department of Pathobiological Sciences

191 Skip Bertman Drive, Room 3315

Baton Rouge, LA 70803

wbeavers@lsu.edu

Running title: ATP sufficiency protects *S. aureus* from PUFA killing

**Supplemental Figure 1. Lipid peroxidation of polyunsaturated fatty acids.**

**Supplemental Figure 2. Bicarbonate repolarizes the membrane and increases cellular ATP, protecting *S. aureus* from AA killing. A.** Membrane potential was assessed in JE2 treated with or without CCCP, sodium bicarbonate, and AA. Fluorescence measurements (red, ex: 488 nm, em: 595 nm; green, ex: 488 nm, em: 530 nm) monitored the ratio of the dye inside and outside of the cell. Data are mean ± S.D. acquired in biological triplicate. Statistical significance was determined by One-way ANOVA, where * = *P* < 0.05, *** = *P* < 0.001, and **** = *P* < 0.0001. **B.** Cellular ATP levels were measured in JE2 treated with or without CCCP, sodium bicarbonate, and AA. Data are mean ± S.D. acquired in biological triplicate. Statistical significance was determined by One-way ANOVA, where ns = *P* > 0.05, * = *P* < 0.05, ** = *P* < 0.01, and **** = *P* < 0.0001. **C.** JE2 was combined with or without AA or sodium bicarbonate and incubated for 17 h. Killing was assessed by dilution plating onto solid medium following the incubation. Data are mean ± S.D. for six biological replicates per condition. Statistical significance was determined by One-way ANOVA, where ns = *P* > 0.05 and **** = *P* < 0.0001.

**Supplemental Figure 3. PUFA killing of *S. aureus* small colony variants is mediated through autoxidation. A.** *menD* overnights were diluted and treated with or without 25 μM LA, AA, or DHA. Growth was monitored by optical density at 600 nm for 48 h. Data are mean ± S.E.M. for measurements acquired for six biological replicates per condition. **B.** The time for each curve in Supplemental Figure 3A to reach OD_600_ = 0.13 (dashed red line) compared to the *k*_p_ for each fatty acid was plotted. Simple linear regression calculated the R^2^ value to determine the correlation between fatty acid-induced growth delays and the *k*_p_ for each fatty acid. Data are mean ± S.D for measurements acquired for six biological replicates per condition.

**Supplemental Figure 4. The *S. aureus* small colony variant, *menD*, is resistant to paraquat growth inhibition. A.** JE2 overnights were diluted and treated with a range of paraquat concentrations. Growth was monitored by optical density at 600 nm for 24 h. Data are mean ± S.E.M. for measurements acquired for six biological replicates per condition. **B.** *menD* overnights were diluted and treated with a range of paraquat concentrations. Growth was monitored by optical density at 600 nm for 24 h. Data are mean ± S.E.M. for measurements acquired for six biological replicates per condition.

**Supplemental Figure 5. Pyruvate increases cellular ATP, but is rapidly consumed, in *menD*.** Cellular ATP levels were measured in JE2 or *menD* grown in TSB with sodium chloride (vehicle) or sodium pyruvate (pyr) for 2.5 h or 5 h. The data displayed are the ratios of ATP in pyruvate treated cells per ATP in vehicle treated cells at the indicated time points. Data are mean ± S.D. acquired in biological triplicate. Statistical significance was determined by One-way ANOVA, where ns = *P* > 0.05 and **** = *P* < 0.0001.
